# Enhancing the usability of low-cost eye trackers for rehabilitation applications

**DOI:** 10.1371/journal.pone.0196348

**Published:** 2018-06-01

**Authors:** Rahul Dasharath Gavas, Sangheeta Roy, Debatri Chatterjee, Soumya Ranjan Tripathy, Kingshuk Chakravarty, Aniruddha Sinha

**Affiliations:** Embedded Systems & Robotics, TCS Research and Innovation, Tata Consultancy Services, Kolkata, India; University of Tübingen, GERMANY

## Abstract

Eye tracking is one of the most widely used technique for assessment, screening and human-machine interaction related applications. There are certain issues which limit the usage of eye trackers in practical scenarios, viz., i) need to perform multiple calibrations and ii) presence of inherent noise in the recorded data. To address these issues, we have proposed a protocol for one-time calibration against the “regular” or the “multiple” calibration phases. It is seen that though it is always desirable to perform multiple calibration, the one-time calibration also produces comparable results and might be better for individuals who are not able to perform multiple calibrations. In that case, “One-time calibration” can also be done by a participant and the calibration results are used for the rest of the participants, provided the chin rest and the eye tracker positions are unaltered. The second major issue is the presence of the inherent noise in the raw gaze data, leading to systematic and variable errors. We have proposed a signal processing chain to remove these two types of errors. Two different psychological stimuli-based tasks, namely, recall-recognition test and number gazing task are used as a case study for the same. It is seen that the proposed approach gives satisfactory results even with one-time calibration. The study is also extended to test the effect of long duration task on the performance of the proposed algorithm and the results confirm that the proposed methods work well in such scenarios too.

## 1 Introduction

In recent years, eye tracking is gaining huge importance for diagnosis and screening [1] of various medical conditions, home-based rehabilitation [2] and human-computer applications [3] due to its unobtrusive nature. Eye tracking is also an important method for analyzing different cognitive functions [4] associated with variety of tasks like reading, writing, visual searching, driving and so on. Non-invasive eye trackers can also be used to study infant cognition [5] in unconstrained, naturalistic environment. However, the accuracy or the robustness of such applications mostly relies on the quality of the data collected. Noisy eye movement data leads to misleading interpretations and outcomes.

**Fig 1:**
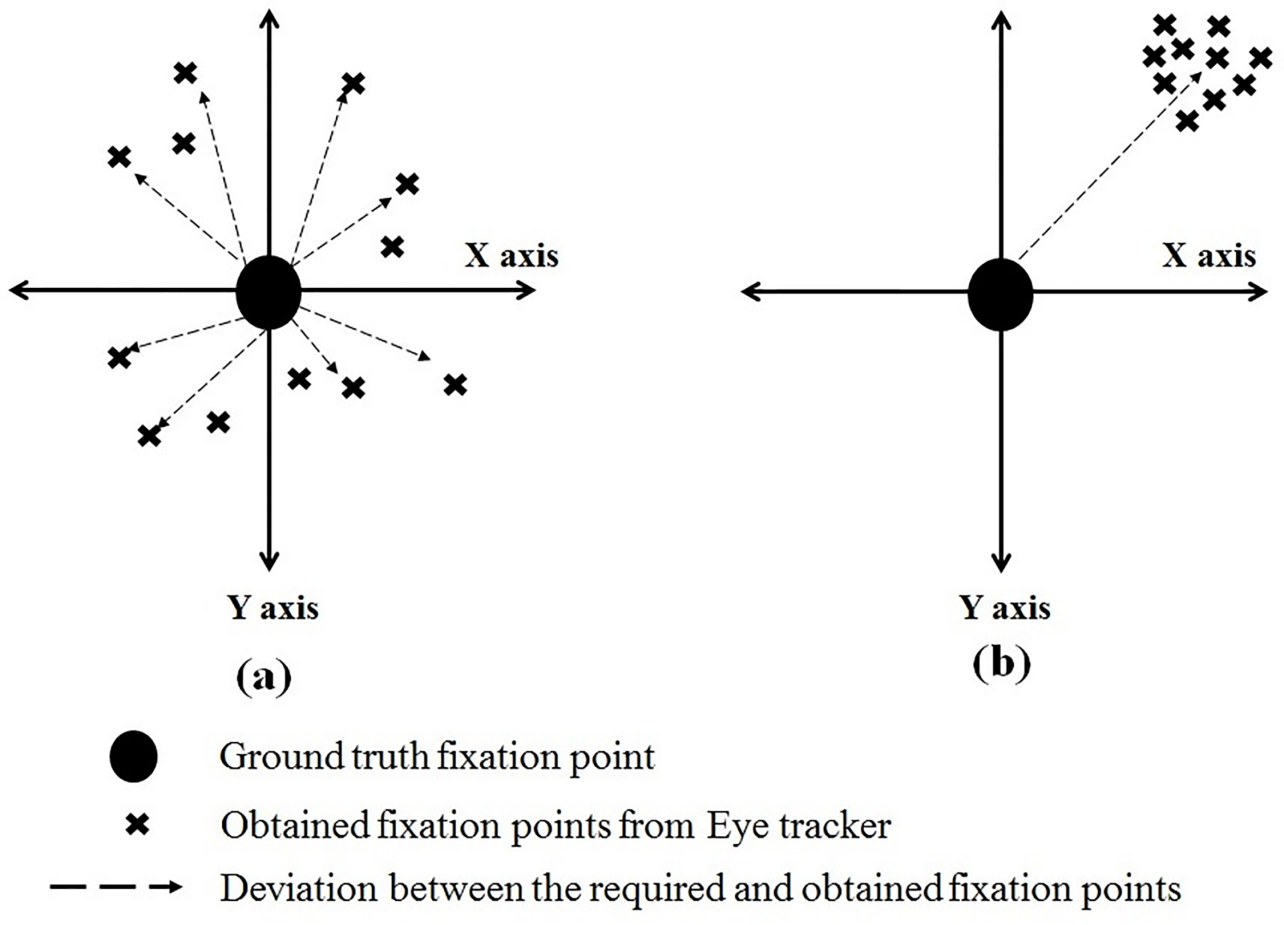
Two types of errors associated with eye trackers, (a) Variable and (b) Systematic error.

Statistics shows that about 8% of world’s total population is the aged population [6], most of which are suffering from some ailments leading to cognitive decline [7] affecting their occulomotor response. Lagun et al [8] showed that Visual Paired Comparison (VPC) task usually provides insights to memory impairments associated with mild cognitive impairment which often progresses to Alzheimer’s disease. Saccadic eye movements can also be used to quantify motor impairments in Parkinson’s disease (PD) [9]. Regular monitoring of eye movement definitely plays a crucial role in assessing cognitive states of such patients. On the other hand, in order to be an ideal choice for home-based rehabilitation applications, the eye tracking device should be portable, easy to use and most importantly affordable. However, such low-cost devices are majorly low in resolution, thereby compromising on the quality of the data [10] recorded. This is usually handled to some extent through a calibration phase that needs to be performed at the beginning of each session and most of the times, in between the experiments too [11]. This is termed as multiple calibration, which is a cumbersome and repetitive process. Moreover, achieving good calibration is a major challenge for patients (with Stroke, Parkinson’s disease, Dementia, Schizophrenia etc.) and infants [10] as they lack the patience and capability to gaze on a fixed point for longer duration. The process also leads to exhaustion or loss of engagement during the actual task that is performed after the calibration phase. Hence, there is a need for establishing a one-time calibration protocol for experiments/tasks targeted for the above discussed participant groups. Another major problem associated with these low-cost eye trackers are the presence of huge inherent noise in the recorded data. Even if we somehow manage to achieve a good calibration score, the quality of data acquired after such sessions is highly susceptible to inherent noise which is a result of head movements, glitches in the eye tracker sensor algorithms, lightning conditions, and so on. In addition to this, the subject-specific variances due to drift, micro-saccades, tremors, etc. are also present in the collected eye gaze data. These issues actually gives rise to certain noise in the collected eye tracker data which can be broadly classified into 2 major classes – variable and systematic errors [12, 13] as shown in Fig 1. The former refers to the dispersion of the gaze coordinates for a given target (Fig 1(a)) and the latter refers to the drift from the target location (Fig 1(b)). These errors are present irrespective of single or multiple calibrations; but the degree of systematic error is larger in the former in comparison to the latter [14]. Variable error is indicative of the lack of precision and the systematic error is indicative of the lack of accuracy [12]. The non-systematic/variable errors are mainly related to physiological characteristics of a participants’ eye and fatigue; and head motion [15]. Changes in screen illumination, participants’ ethnicity and operating distance from the screen [16], etc. also add to the degradation of the eye tracker accuracy. High-end eye tracking systems like Tobii also suffers from such errors. Efforts to denoise Tobii are reported in [14]. However, such high-end eye trackers are not mass-deployable for home-based rehabilitation owing to their high cost; whereas, in low resolution eye trackers, the amount of system generated noise is greater than the natural (inherent) noise. Without the removal of these errors, it is difficult to use eye trackers for practical applications, especially in human-computer interaction (HCI)-based clinical applications [17–19].

Thus, it can be concluded that the two major challenges that reduce the usability of low-cost eye trackers in various medical or rehabilitation applications are as follows: 1) need for multiple calibration and 2) inherent variable and systematic errors. The present study proposes an algorithm which takes care of the noises associated with eye trackers and also a protocol in order to avoid the overhead of multiple calibration is devised.

The present work is the detailed algorithmic version of the simple eye tracking noise cleaning approaches that we proposed earlier [20] with a larger population set, additional metrices to study the performance of the proposed algorithms on variable and systematic error correction, detailed analysis of the supervised and unsupervised approaches and the study of the effect of longer duration tasks on the proposed approaches.

## 2 Related work

Eye trackers are mainly categorized into 2 types based on their design features, namely remote (nearables [21]) and wearables (head mounted [22]). Each one of these comes with its own set of advantages and disadvantages. For instance, the nearable ones are unobtrusive in nature but are less efficient in comparison to the wearables. Also, the participants become more cautious about the sensors that they are wearing during the actual experiment. One of the most robust wearable eye trackers uses contact lens, wherein the tracking mechanism is embedded into the lens [23]. However, it is costlier in comparison to other eye tracking devices and also its complexity makes it less user friendly. Electro-occulogram (EOG)-based eye tracking is yet another popular means of detecting eye movements by acquiring the minute changes generated by the corneal-retinal potentials of the eyes [24]. This method is highly vulnerable to various electrical noises and drift errors. Also, the complex circuitry consisting of wires and gel-based electrodes make it less appealing for practical scenarios. For large-scale deployment, the device should be low-cost and unobtrusive in nature. Video-based and infrared-based eye trackers are popularly used as unobtrusive means of eye tracking. Video-based eye tracking is a popular technique [25] in which a camera focuses on the eyes and the eye ball movements are recorded. One variant of this as proposed by Zhang et al. [26] is basically an appearance-based method which does not require calibration, but the accuracy of detection is highly dependent on ambient lighting conditions; thereby degrading its performance in real time scenarios. Basilio et al [27] proposed a calibration free method but it faces challenges in real life applications due to head and body movements. The accuracy of the method is less due to the following reasons: 1) severe lighting conditions owing to the head movements towards bright areas of the environment; 2) distortion due to wireless transmission of the video data and 3) absence of user calibration. Therefore, the accuracy of video-based eye trackers is compromised due to head movements, which are prevalent during longer duration experiments, thereby limiting the usage in short-term experimental sessions only. The infrared-based methods are less complex, cost-effective [28] and un-obtrusive in nature [29]. Subject-specific attributes like eyelashes covering the pupil, eye glasses or contact lenses, physiological characteristics of the eye like additional dark spots on the iris, interferes with the pupil detection algorithms [30]. In addition to these, the factors like changing illumination, recording errors, motion blur, rapidly changing illumination due to the fast movement of the participant (for instance, while driving) also adds to the errors in pupil position measurement [30]. There have been attempts to use low-cost infrared eye trackers in HCI-based applications [3]. These low-cost devices are basically lower in resolution and calibration phase plays a major role in determining the quality of the data. Multiple calibrations seem an attractive means but accomplishing it, is often cumbersome and exhaustive process [10]. Bereft of the modes of the calibration, the inherent noise namely, variable and systematic, poses major challenge for using eye tracker data.

In general, filtering-based approach is used for excavating the variable error. Most of the filtering-based approaches remove the abrupt fluctuations in the gaze data, thereby smoothing the overall signal. Some approaches design low pass filters, as suggested by Olsson et al. [31], which use both offline and online filtering to remove the noise. In the offline approach, the fixation data is extracted from the raw noisy data using sliding window approach, whereas for online approach, it estimates the filtered data by considering the mean of previously estimated position data. Many researchers proposed various techniques to compute the window size [32, 33] to estimate the fixation data. An advantage of this approach is that they allow increasing the window size depending on the application. In [34], the authors proposed a hybrid filter. It is composed of several linear FIR (Finite Impulse Response) sub-filters and finally, it performs a median filter operation over the outputs of sub-filters. The advantage of this approach lies in the ability to preserve the sharp fluctuations by attenuating the noise to some extent, whereas the fluctuations in the signal are suppressed considerably in linear low-pass filtering methods. The accuracy of these methods depends on several parameters, such as window length, fixation detection threshold, which in general are difficult to estimate as these parameters largely depend on the magnitude of the noise present at any instant of time. Some works suggest using the Kalman filter for denoising the data. It eliminates the need for storing previously observed data at each step of the filtering process. Sauter et al. [35] have proposed eye-movement detection using Kalman filter. Many authors [36] [37] have extended the Kalman filter for identifying different eye movements based on their applications. For example, in [37], the authors used Kalman filter to classify different types of eye movements and to reduce sensor lag through eye movement prediction. On similar grounds, Komogortsev et al. [38], used an attention-based Kalman filter, which aims at reducing the noise in addition to minimizing the delay between eye gaze-based systems and displayed data for designing an interaction model based on eye movement language token.

One of the popular methods of handling the systematic error is based on the concept of extraction of ‘required fixation location’. If the region on screen where the participant is gazing is known, then correcting the discrepancy in the gaze data and the ground truth is handled by estimating the amount and the direction of the drift [12]. The major shortcoming of this approach, however, lies in the fact that the error signature need not be constant throughout the experiment [39]. The error varies with sessions due to head movements, fatigue of the participant, screen illumination, changes in the distance from the screen and the ethnicity of the participants [16], etc. Also, the concept of ‘required fixation location’ does not apply in most of the real life scenarios, i.e. it is not always possible to know the ground truth of the gaze data. Another major approach is based on the principle of ‘closest stimulus’ [14] which applies the principle of annealed mean shift algorithm. This method suffers severely when the fixation has many stimulus points around it and the closest point might not be the desired target location. Also, the fact that calibration error is sometimes location dependent on the screen, the performance of this method is thus compromised [39]. Vadillo et al. [39] have proposed a linear transformation (LT) algorithm to correct the systematic error by using the concept of ‘probable fixation location’, which is more likely to be prevalent in practical cases. However, this method too does not retain the nature of the drift, rather it deals with the discrepancy between the target and the gaze data.

In case of human eye movements, there are inherent noises due to drift, micro-saccades, tremors, etc. However, along with these artifacts, there are noises like the variable and the systematic errors in the eye tracker data. In low resolution eye trackers, obtaining clear demarcation between the device imposed and naturally generated error, is difficult. Moreover, our aim in the current use-case is to handle fixations, and not to detect micro-saccades. The naturally generated noise is constant bereft of the quality of the eye tracker. But, in low resolution eye trackers, the characteristics of device generated noise and the natural noise closely resembles each other. Without the removal of these errors, it is difficult to use the fixation information in controlling HCI-based applications.

## 3 Design of stimulus

In the present study we have designed i) a set of stimulus to be used for calibration and ii) two test stimulus for evaluating the accuracy of the proposed methodology with respect to noise removal.

### 3.1 Calibration stimulus

Calibration is an important phase in eye tracking to collect data in order to map the coordinates of the pupil movements in the eye-video to that of the coordinates in the stimulus space. Hence, the challenge is to gather data from as many known locations as possible but with least mental effort on the participant and ensuring sustained attention on the target [40]. Blignaut [40] proposed a calibration scheme which involves collecting the data during smooth movements of the eye, termed as smooth pursuit. Thiago et al [41] proposed a dynamic calibration scheme called *CalibMe* which uses eye movements for collecting data during calibration. This method allows free head movements during calibration. In this work, we have used a simple calibration phase, which has static calibration points appearing at pre-defined positions and then it moves smoothly by generating smooth pursuits. However, we haven’t used the smooth pursuits for the calibration and the readers are free to use any of the above discussed calibration schemes depending upon their applications and target participants.

**Fig 2:**
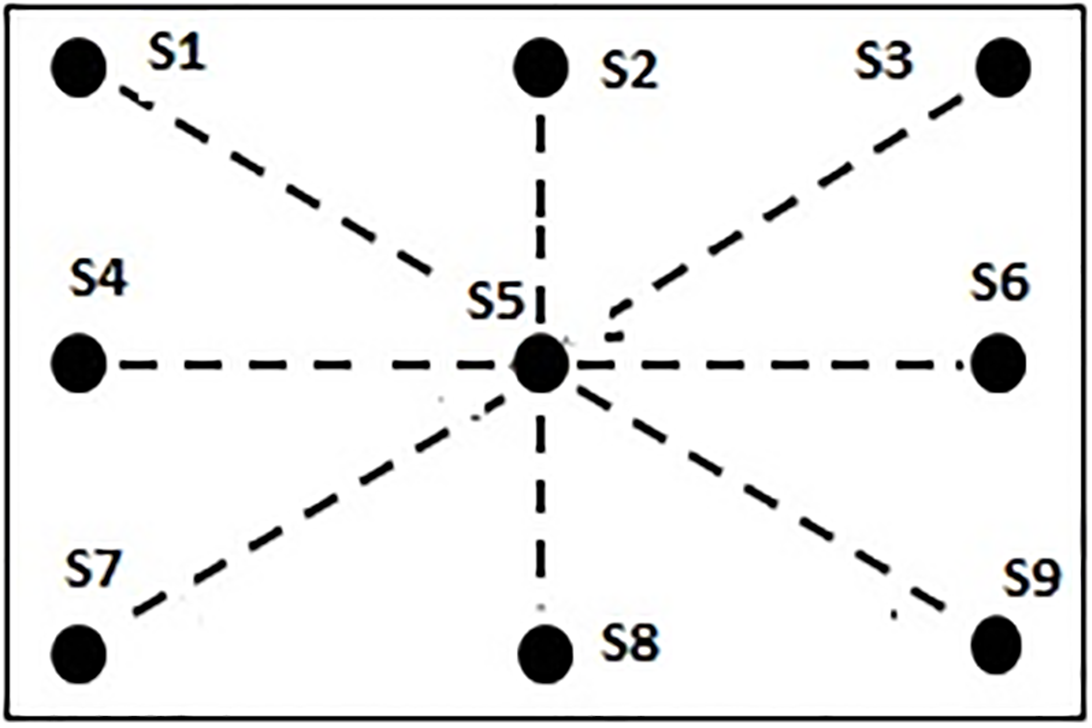
Schema of the designed calibration stimulus with the calibration point having diameter 0.657° and moving at a constant speed of 1.92°/sec on the screen.

For the calibration purpose, 4 sets of stimuli are designed and developed using Pygame [42] (Fig 2). The stimulus consists of a tiny ball, having a field of view (FOV) of approximately, 0.657°, moving at a constant speed of 1.92°/sec on the screen. The FOV of 0.657° corresponds to the tiny ball having a diameter of 20 pixels viewed at a distance of 60 cm. In order to cover the entire screen during the calibration phase, the ball moves in horizontal, vertical and in 2 diagonal directions as shown in Fig 2. During each of these movements, the ball stops for 5 seconds at nine positions, shown as dark spots (*S*1 through *S*9) in the Fig 2, and then it moves again along the path shown as dotted lines. The size of the ball is deliberately kept small so that the participants can easily fixate at the center of the ball. The participants are supposed to gaze carefully at the ball while their gaze data are logged using the eye tracker. In total, we have 9 static points, *S*1 through *S*9 whose corresponding fixations are used for noise correction.

### 3.2 Test stimulus

The test stimulus is derived from standard psychological tests and redesigned in order to test the robustness of the proposed algorithms. The test stimulus needs to be designed in such a way that it covers a broader spectrum of psychological test batteries. In this work, two stimuli are designed to test the accuracy of noise correction—1) recall-recognition (RR) [43] and 2) number gazing task (NG) (analogous to the Digit-Symbol Substitution Test (DSST) [44]) using Pygame package. A summary of the stimulus is provided in Table 1. It is to be noted that the systematic and variable errors are independent of the stimulus. The NG task contains the stimulus points (numbers through 1-9) only in a single row, whereas, the RR task presents the stimuli (words) in multiple rows. Variation in the inter-stimulus spacing (horizontal gap between 2 numbers) in the NG task and number of words in the RR task, yield different set of challenges for handling the noise.

**Table 1:** Details of the test stimulus used for the study.

	RR task	NG task
**Task**	(i) A list of 6 words is shown first for 30 seconds (ii) Next, a new list of words is shown (iii) Identify words from the new list those match with the first list (iv) Gaze at the word for 2 seconds and then click on that word (v) Repeat the steps (iii) and (iv) for all such matching words	(i) A sequence of 4 numbers (pin) is given (e.g., ‘1234’) beforehand (ii) A sequence of 9 numbers is shown on the screen (iii) Gaze each number on the screen in the sequence as given in the pin (iv) Click on the number after gazing for seconds (v) Repeat the steps (iii) and (iv) for each number in the pin
**Entities**	Words taken from NIMHANS neuropsychological test battery [45]	Numbers (1-9)
**Arrangement of entities**	Multiple rows, 2 columns	Single row, 9 columns
**Variations in the task**	Difference in number of words in a column	Difference in inter-digit spacing between the 9 numbers with FOVs 3.28°, 2.29° and 1.642° (100, 70 and 50 pixels), respectively
**Font size**	48 pixels	Font sizes used are 50, 35 and 25. The ratio of inter-digit spacing and font size is kept constant to 2:1

**Fig 3:**
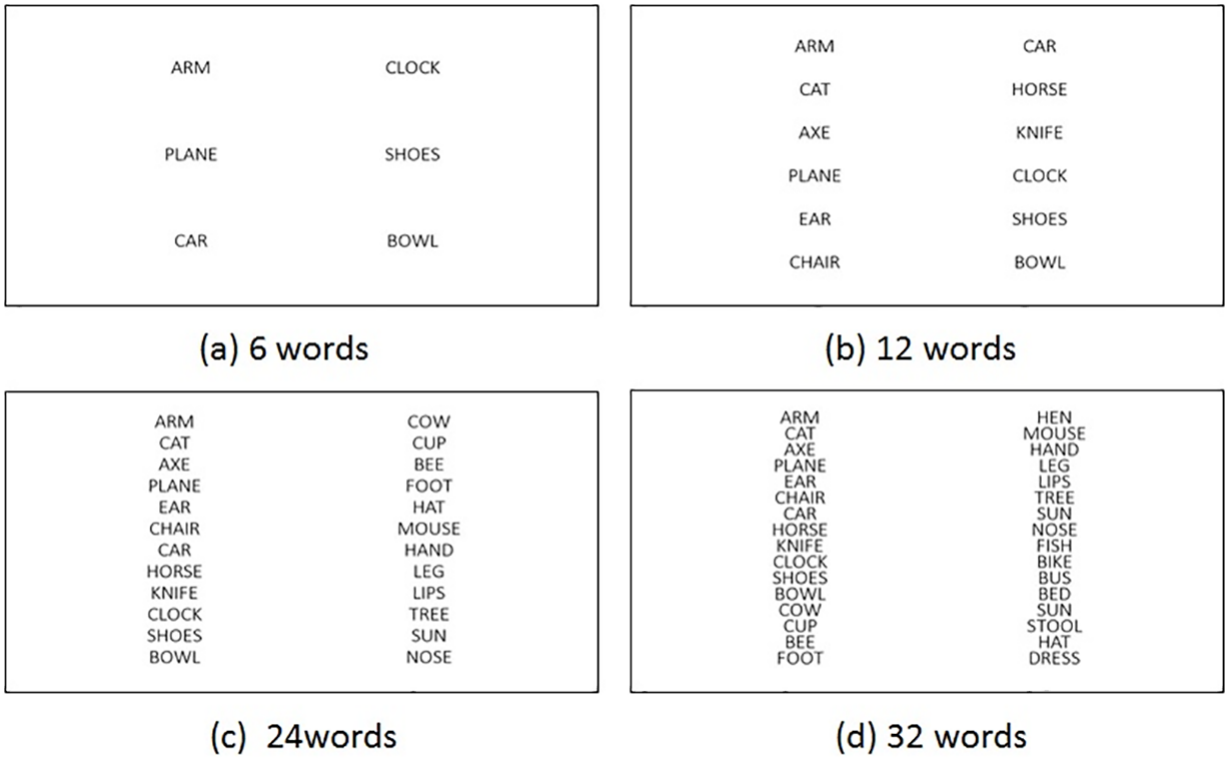
Designed recall-recognition (RR) test (a) 6 words for recall (3 words/column); and (b) 12 words (6 words/column) (c) 24 words (12 words/column) and (d) 32 words (16 words/column) for recognition.

In the RR task, initially a list of 6 words is shown to the participants and they are instructed to memorize the words. Next, a new list of words is shown to them. Some of the words from the first list are also present in the second list. The participants are asked to recognize those words and click on them. The font size of the words is selected to be 48 pixels (1.57°) [46] and the words are presented in a 2 column format. In order to evaluate the performance of noise cleaning algorithms (gaze tracking), the number of words per column is varied from 6 to 16. Fig 3 shows a snapshot of the designed task. The words have been chosen from National Institute of Mental Health and Neurosciences (NIMHANS) neuropsychological test battery [45].

**Fig 4:**
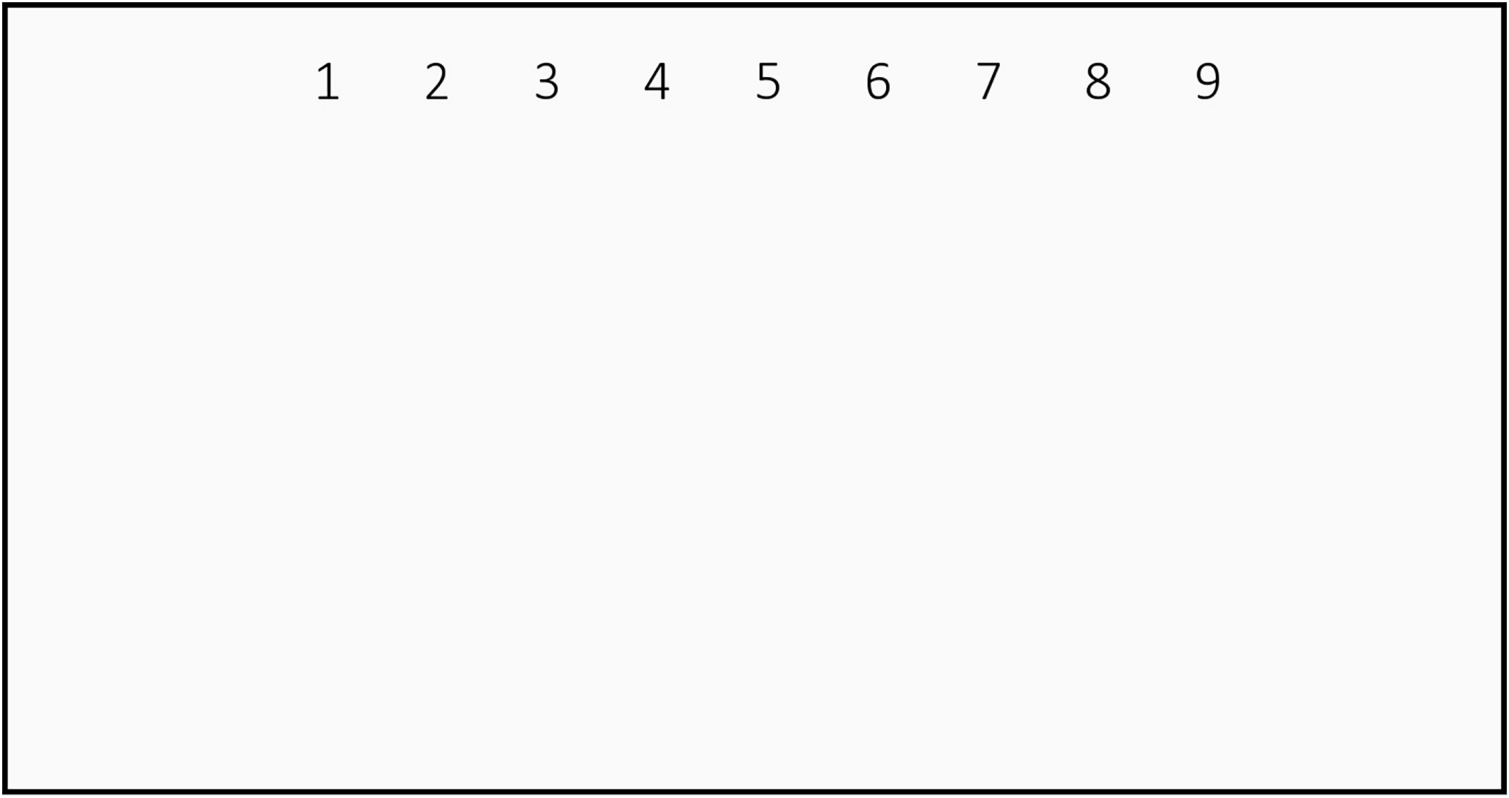
Number gazing (NG) task for inter-digit spacing of 100 pixels.

The second test stimulus consists of a NG task wherein, a lookup table of 9 digits (1 to 9) is shown at the top of the screen as shown in Fig 4. The participants are instructed to gaze at 4 digits, one after the other, in a predefined sequence (as communicated by the instructor/experimenter) before starting the task. The inter-number spacing (*S*), and the font size (*f*) are varied in each trial keeping the *S*/*f* ratio constant. Three trials are conducted with inter-digit spacing of 100, 70 and 50 pixels, respectively. The font size *f* for the 3 different spacings are respectively, 1.675°, 1.182° and 0.854°.

After gazing at a particular word or a digit, the participant is instructed to click on the same. The coordinates and timestamps of the click event are logged in order to segment the corresponding eye gaze data for further analysis.

The underlying motivation for the selection of these test stimuli are as follows. Recently various medical applications are being developed keeping in mind the overall mental/cognitive well-being. On the other hand, alternative communication aids for patients with neuro-motor disorders, controlling wheel chair or various devices through human computer interfaces [47], gaze tracking-based applications for patients suffering from Autism [48], and also various standard cognitive assessments like SDMT [49], trail making task, etc. are some of those kinds of applications. In most of these applications, attention, memory retention, working memory, etc. are important aspects. RR test used in the present study is a standard psychological test, which is used to assess higher order cognitive functions, like memory retention capacity and attention. On the other hand, the NG task additionally involves the usage of working memory in order to correctly sequence the fixations on the given order of numbers. Thus the stimuli used, closely resembles the tasks that are performed by psychologists to assess cognitive functionalities of an individual.

## 4 Methodology

In the present study, we propose a novel approach of handling errors by using the nature of the drift or the systematic error from the neighborhood regions. Along with this, we have applied the principle of ‘*n*-nearest stimulus points’ and have adopted unsupervised techniques. In addition to this, a novel supervised method based on the concept of ‘*n*-nearest calibration points’ is applied. The essence of ‘*n*-nearest’ over ‘closest stimulus point’ [14] and ‘*n*-nearest’ over ‘closest calibration point’ is introduced along with inverse weighing function-based approximation. The accuracies of all these approaches are compared.

**Fig 5:**
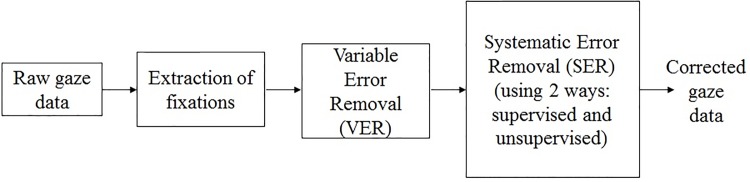
Block-diagram of the proposed method.

This section explains the noise removal approaches adopted in the present study. It also explains the methodology adopted for generating corrected gaze data from raw eye tracker data. The overall process is shown in the Fig 5.

**Fig 6:**
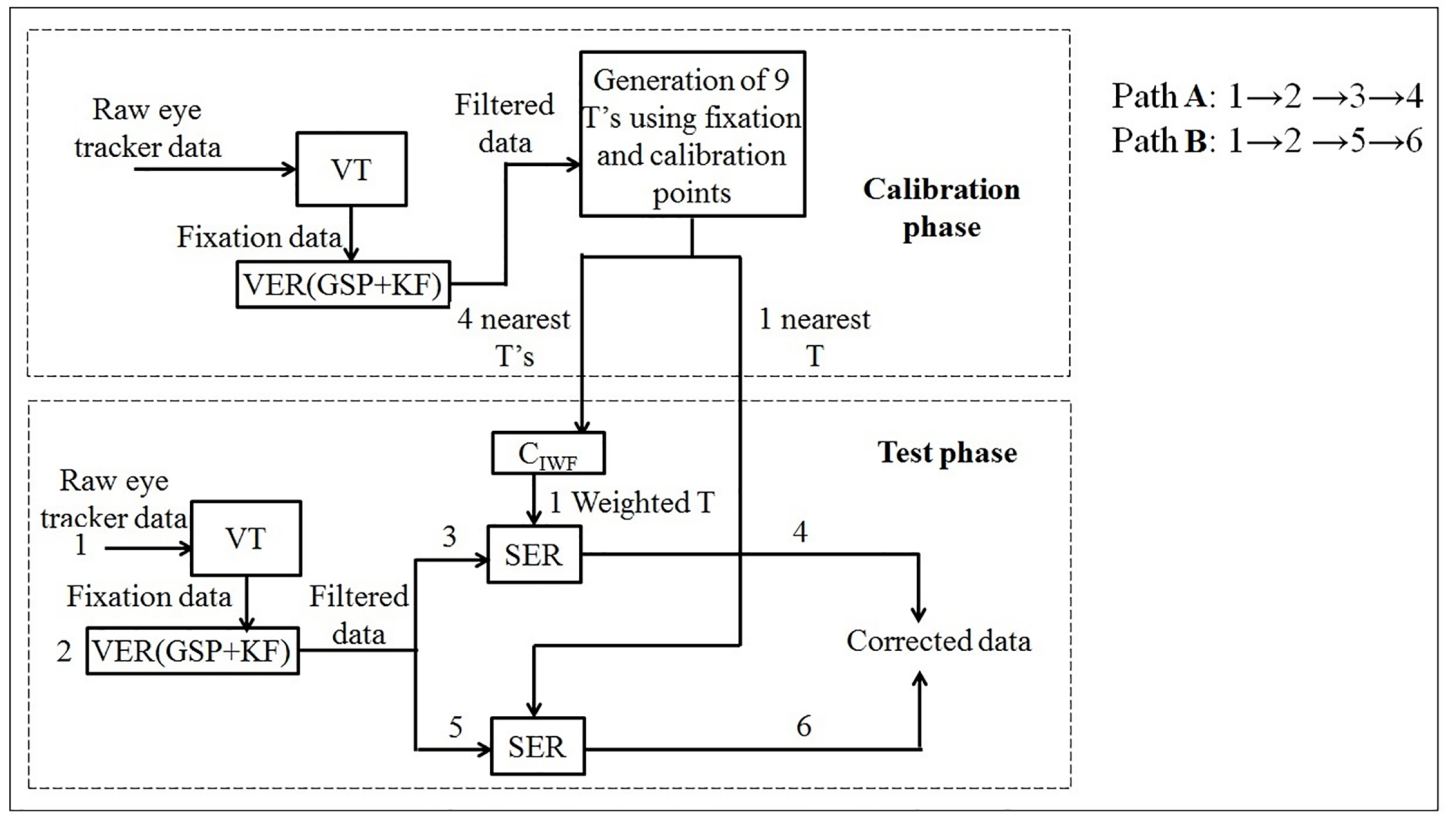
Supervised approach for data correction.

**Fig 7:**
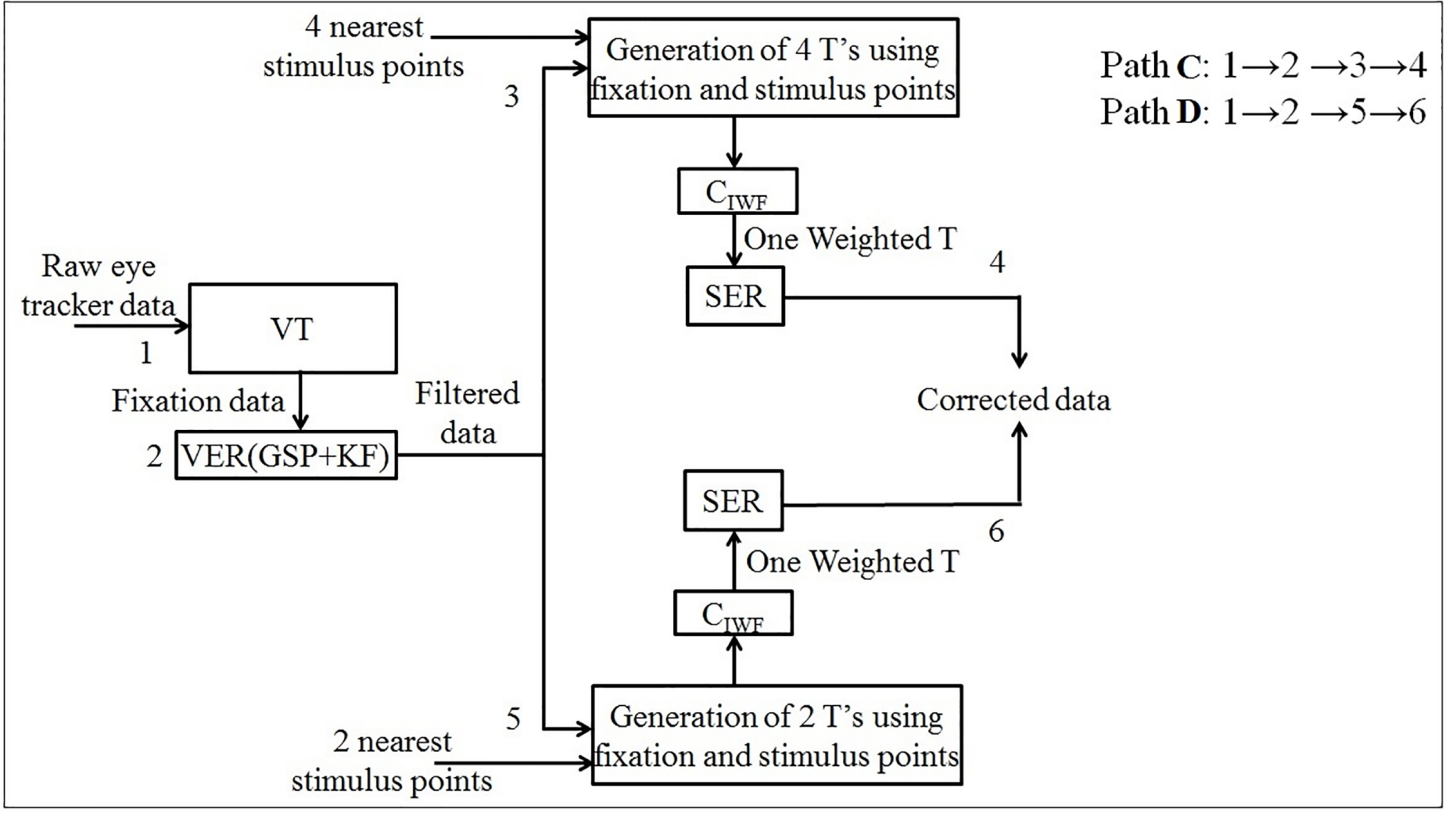
Unsupervised approach for data correction.

First the fixation data are extracted from the raw (unprocessed) eye tracker data. Next, the variable error is filtered from this data. Finally, the systematic error is removed to get corrected gaze coordinates using both supervised and unsupervised approaches. Each of these approaches are explained in detail in Figs 6 and 7.

### 4.1 Extraction of fixations from raw eye tracker data

Eye movement data can be classified into 2 major classes, i.e., fixations and saccades. Many works pertaining to the classification of eye tracker data into these classes exists [50], [51, 52]. Enkelejda, et al [51] proposed the usage of low resolution eye tracker in approximating the clusters of fixation to a region of interest (ROI) using online bayesian learning. However, even in a given fixation chunk, the variable error persists. We have extracted the fixations from the raw eye tracker data using the velocity threshold-based method (VT) as explained in [50]. Eye gaze data usually consists of fixations and saccades. The data points lying above a threshold velocity are treated as saccades and the rest are categorized as fixations. As suggested in [53], we have used the velocity threshold value as 20.

### 4.2 Variable error removal (VER)

To handle the fluctuations or the variable error in the fixation data, we have made a survey of various filtering approaches available in literature. In the present study, we have used the graph signal processing (GSP) [54] and Kalman filter (KF) [55] for removing the variable error. The application of GSP and KF on the raw eye gaze data is explained as follows:

#### 4.2.1 Graph signal processing-based signal cleaning

During the data capture, the eye tracker captures the noisy (*x*, *y*) gaze coordinates on a plane (monitor) reported by the eye tracker and can be represented as Eq (1),

$$\begin{array}{c} S={\left[\left(x_{1},y_{1}\right),\left(x_{2},y_{2}\right),...,\left(x_{n},y_{n}\right)\right]}^{T} \end{array}$$ (1)

where *n* is the number of samples in the signal. In our experiment as we focus on a single position on the screen, the eye gaze signal should return a single coordinate. However, *S* fluctuates due to the presence of variable error. Sometimes, the fluctuations are not mere oscillations around the actual position, rather these are far away from it. Hence, the denoising algorithm needs to be designed in such a way that it can handle those abrupt changes and produce a smooth signal, which is close to the actual eyeball location. Hence, GSP is suitable for this application as it smoothens the signal in accordance with the underlying graph structure, unlike other low pass filtering (LF) methods [31]. In order to perform the denoising, *S* is divided into a number of non-overlapping windows of length *L*(≤ *n*) and then GSP-based denoising is applied on each of these windows separately. In order to do so, first a graph signal *G*(*V*, *E*, *A*) is formed, which is characterized by a set of vertices *V*, set of edges *E* and an adjacency matrix *A*, which stores the weighted connection between the vertices. In our case, *V* is formed by taking the coordinates (*x*_*i*_, *y*_*i*_) in a particular window. The connection is formed by keeping all the vertices pairs between which an edge exists. The edges are formed if the Euclidean distance between the two vertices *n* and *m* is less than a threshold value *th* (empirically taken as 1) and the set of edges can be expressed as,

$$\begin{array}{c} E=(n_{i},m_{j}) \end{array}$$ (2)

In this type of range-based searching, each of the vertices has different number of neighbors in a particular window, which introduces dynamicity in the graph formation and provides an edge over other filtering methods. The weighted adjacency matrix *A* is constructed by putting weights on edges depending on closeness measure between the two vertices. Closeness between two vertices is measured by the Euclidean distance between those 2 vertices. Hence, the weight of the connection between vertices *n* and *m* is defined using a Gaussian kernel for a constant *θ* as shown in Eq (3),

$$\begin{array}{c} {\vec{C}}_{B}=\left\{ \begin{array}{cc} exp(\frac{-[dist[n,m]]}{\theta^{2}}), & \text{if}(n,m)\in E\\ 0, & \text{otherwise} \end{array}\right. \end{array}$$ (3)

In our study, *θ* is chosen to be 1. The graph signal *G* formed in each window is corrupted by variable noise and can be written as,

$$\begin{array}{c} G=t+e \end{array}$$ (4)

where *t* is the clean signal and *e* is the noise added to it. In order to obtain the clean signal that is close to the original signal as well as smooth, a multi-objective optimization can be formed in a quadratic form as,

$$\begin{array}{c} argmin_{t}\left(\frac{1}{2}{\left||t-G|\right|}^{2}+\alpha \frac{1}{2}{\left||t-At|\right|}^{2}\right) \end{array}$$ (5)

Here, *α* controls the amount of smoothness desired in the estimated *S*_*t*_. This stated optimization problem can be solved by setting the first derivative of Eq (5) to zero and the closed form solution can be derived as,

$$\begin{array}{c} \frac{1}{2}\frac{\partial }{\partial t}\left({\left||t-G|\right|}^{2}+\alpha {\left||t-At|\right|}^{2}\right)=0 \end{array}$$ (6)

$$\begin{array}{c} \frac{1}{2}\frac{\partial }{\partial t}\left({\left(t-G\right)}^{*}\left(t-G\right)+\alpha t^{*}{\left(I-A\right)}^{*}\left(I-A\right)t\right)=0 \end{array}$$ (7)

$$\begin{array}{c} S_{t}={\left(I+\alpha {\left(I-A\right)}^{*}\left(I-A\right)\right)}^{-1}G \end{array}$$ (8)

where * is Hermitian of the matrix. The solution stated in Eq (6) denoises the graph signal in each window as shown in Eq (8). The formation of the graph is dependent on the size of the window, which can be chosen judiciously. A bigger window provides a smoother signal which is more influenced by the abrupt fluctuations present in that window, whereas smaller windows fail to smooth the signal efficiently. Here, we have heuristically taken *L* as 10. The most expensive step in Eq (8) is the inversion of the matrix (*I* − *A*). In our case the size of A is only of 10 × 10, which makes the (*I* − *A*) inversion affordable in terms of computation. The pseudocode is provided in Algorithm 1.

**Algorithm 1:** Pseudocode for Graph Signal Processing based signal cleaning

GSPfiltering(*S*_*x*_, *S*_*y*_) timeseries of eye gaze position;

**Input**      :Timeseries of eye gaze coordinates *S*

**Output**     :GSP filtered eye gaze data *S*_*t*_

**Initialization**   :Window length L = 10, *θ* = 1, *α* = 5, *th* = 1

**FOR** each time window k

 **Graph G(V, E, A) Formation**    :

     Edge *E* formation by finding the eye gaze positions whose euclidean distances fall inside the unit circle (*th* = 1)

     Compute the closeness measure as euclidean measure between two connected vertices

     Edge weight *C*_*B*_ is defined as a Gaussian kernel over closeness measure with constant *θ* if there is any edge between two vertices or 0 otherwise

     Adjacency matrix *A* is computed for *G*(*V*, *E*, *A*)

 **Estimation of clean signal (*S*_*t*_) for a window**:

      Filtered signal, *S*_*t*_ = (*I* + *α*(*I* − *A*)*(*I* − *A*)^−1^)*G*

   Loop continue for other windows

#### 4.2.2 Kalman filter (KF)-based signal enhancement

In order to minimize the noise further, we have used KF on the GSP filtered data *S*_*t*_{*S*_*x*_, *S*_*y*_}. The state vectors at time *k* is given by, $\vec{R_{k}}=\left[S_{x_{k}}S_{y_{k}}S_{x_{k}}^{.}S_{y_{k}}^{.}\right]$, where, $S_{x_{k}}^{.}S_{y_{k}}^{.}$ denotes the velocity of eyeball among the *X* and the *Y* directions, respectively. The instantaneous eye movements depend on the prior velocities, i.e. $\vec{v_{j}}=\left\{S_{x_{j}}^{.}S_{y_{j}}^{.}\right\}$ where time (*j* < *k*) and hence we have modeled $\vec{v_{k}}$ as the weighted sum of previous velocities. The dynamic equations that govern the position $\vec{p_{k}}\left(S_{x_{k}}S_{y_{k}}\right)$, for (*x*, *y*) position at instance *k*, of eye gaze data are,

$$\begin{array}{c} {\vec{p}}_{k}={\vec{p}}_{k-1}+T*{\vec{v}}_{k-1} \end{array}$$ (9)

$$\begin{array}{c} {\vec{v}}_{k}=a_{k-1}{\vec{v}}_{k-1}+a_{k-2}{\vec{v}}_{k-2}+a_{k-3}{\vec{v}}_{k-3}+\phi \end{array}$$ (10)

where $T=\frac{1}{f_{s}}$; *f*_*s*_ is the sampling frequency (30 Hz) of the Eye Tribe eye tracker. It is observed that the eyeball velocity follows ARIMA (3, 0, 0) or AR(3) [56] and hence, we have derived the coefficients *a*_*k*−1_, *a*_*k*−2_, *a*_*k*−3_ and *ϕ* from the ARIMA model. It is required that the coefficients are to be derived separately for each participant. The discrete state space model for eye gaze data is given by the linear stochastic difference at time *k* as,

$$\begin{array}{c} {\vec{R}}_{k}=F{\vec{R}}_{k-1}+{\vec{w}}_{k-1} \end{array}$$ (11)

$$\begin{array}{c} \vec{Z_{k}}=H{\vec{R}}_{k}+{\vec{\rho }}_{k} \end{array}$$ (12)

where *F* is the state transformation matrix. The actual observation is made at time *k*. The noiseless connection among the measurement vector ${\vec{Z}}_{k}$ and state vector ${\vec{R}}_{k}$ is designated by *H*. The ${\vec{\rho }}_{k}$ and ${\vec{w}}_{k}$ are measurement and process noise (uncorrelated gaussian noise following zero mean and co-variance of *ϕ*_2_ and *ϕ*_1_), respectively. The Kalman filter corrects the eye gaze data ${\vec{R}}_{k}$ after receiving ${\vec{Z}}_{k}$ (at time *k*) by,

$$\begin{array}{c} {\hat{R}}_{k}={\vec{R}}_{k}+K_{k}\left({\vec{Z}}_{k}-H{\vec{R}}_{k}\right) \end{array}$$ (13)

where *K*_*k*_ is the Kalman gain [57] and ${\hat{R}}_{k}$ is the filtered data at time *k*. The pseudocode is as provided in Algorithm 2.

**Algorithm 2:** Pseudocode for Kalman filtering for denoising the signal

KalmanSmoothing (*S*_*t*_*x*__, *S*_*t*_*y*__);

**Input**    : GSP filtered eye gaze coordinates *S*_*t*_ = (*S*_*t*_*x*__, *S*_*t*_*y*__)

**Output**   : Smooth data ${\hat{R}}_{k}$

**Initialization** : Estimated state vector ${\vec{\hat{R}}}_{k|k-1}^{+}$, state transition matrix F, measurement mapping matrix H, process noise co-variance matrix *ϕ*_1_, measurement noise co-variance matrix *ϕ*_2_, priori state co-variance ${\vec{\hat{P}}}_{k-1}$

**FOR** each time epoch k

 **Prediction** : State prediction based on (*k* − 1)^*th*^ state given *F* and *ϕ*_1_

 **Update**  : Update the posterior mean of state estimate based on the new measurement ${\vec{S}}_{t}^{k}$ given *ϕ*_2_ and *H*. Compute Kalman Gain *K*_*k*_ and update the covariance ${\vec{\hat{P}}}_{k-1}$ and state estimate ${\hat{R}}_{k}$

Loop continue

### 4.3 Systematic error removal (SER) using linear transformation (LT)

The filtered data, $\hat{R}$ is subjected to further processing with spatial transformation in order to remove the systematic error [39]. The method basically uses the separation between the actual and the desired (ground truth) gaze coordinates for generating a 2 × 2 transformation matrix *T*. The best-fitting values of *T* are obtained using optimization routines such as simplex algorithm [39]. Next, this matrix is used to correct the actual fixation data $\hat{R}$ as,

$$\begin{array}{c} \vec{C}=\hat{R}*T \end{array}$$ (14)

where $\vec{C}$ is the corrected gaze data. In ideal case, if $\hat{R}$ exactly matches the ground truth coordinates, *T* would have been an identity matrix. We have applied both supervised and unsupervised approaches for error removal.

In the supervised approach, the systematic error is learnt in the calibration phase in terms of transformation matrix *T*, which is then used in the succeeding test phase (for supervised approach), whereas it is derived directly from the test data set in case of unsupervised approaches, discussed in the following subsections.

#### 4.3.1 Supervised approaches—Paths A and B

The designed supervised approach is depicted in Fig 6.

The fixation data is extracted from the eye gaze data collected in the calibration phase, as explained in section 4.1. Next, the data is subjected to variable error removal as explained in section 4.2. Finally, the transformation matrix *T* is derived for each of the 9 static points (*S*1 through *S*9). Each of the *T*s are evaluated for correctness. We define a correctness measure *M*, given by,

$$\begin{array}{c} M=det(T) \end{array}$$ (15)

Ideally, if the raw data and the ground truth data exactly matches, *T* would be an identity matrix with *M* = 1. We computed *M* for over 200 fixation chunks and a threshold of 0.8 is set empirically. If *M* < 0.8 for any calibration point, then it is rejected and is replaced by the average *T* of 2 nearest calibration points whose *M* value is greater than the threshold. If more than 3 *T*s have *M* less than threshold of 0.8, a fresh set of data are captured for the calculation of transformation matrices for that particular participant.

The matrices derived from the calibration phase are stored and are used to remove the systematic error in the test phase. The proposed method deals with extracting the transformation matrix *T* for a given fixation chunk with centroid *X* from its nearest calibration point *S*. The main principle behind this assumption is that the nature of systematic error for the given fixation chunk is similar to the systematic error seen on the nearest calibration point *S* (during the calibration phase), computed using k-nearest neighbor search algorithm [58]. Path A, as shown in Fig 6 has 4 *T*s based on inverse weighing function ${\vec{C}}_{IWF}$, defined as,

$$\begin{array}{c} {\vec{C}}_{IWF}=\left\{ \begin{array}{cc} \frac{\sum_{i=1}^{N}w_{i}\left(x\right)\vec{u_{i}}}{\sum_{i=1}^{N}w_{i}\left(x\right)}, & \text{if}\;d(x,y)\neq 0\forall i\\ \vec{u_{i}}, & \text{otherwise} \end{array}\right. \end{array}$$ (16)

where $\vec{u_{i}}={\hat{R}}_{k}*T_{k}$ for *k* = 1 to 4 nearest calibration points; *N* = 4 nearest neighbors; $w_{i}\left(x\right)=\frac{1}{d{\left(x,y\right)}^{p}}$ where, *d* is the Euclidean distance between the centroids of the fixation data *x* and the calibration point (any one among the points *S*1 through *S*9) and the value of *p* is set to 2. The weight *w* is normalized by dividing each of the 4 weights by the sum of the total weight. The weights are inversely proportional to the square of the distance, which implies that the corrected gaze data is mostly influenced by the nearer neighbors. Nearer the point, more the force applied to pull the point towards the calibration point. For path B, as shown in Fig 6, only the transformation matrix corresponding to the most nearest calibration point is taken into account for correcting the systematic error. Hence, in the current supervised approach, we have used either 4 and 1 nearest calibration points. The reason behind using 4 points is to check the influence of systematic error across the screen (i.e. in terms of magnitude and direction of the drift). In contrast to this, we have checked the performance of the filtered fixation data against 1 closest calibration point, which incorporates the nature of the systematic error corresponding to that particular point only. The psuedocodes for paths A and B are provided in Algorithms 3 and 4, respectively.

**Algorithm 3**: Pseudocode for Path A

**Input**   : Raw Gaze data, *S* = (*S*_*x*_, *S*_*y*_)

**Output**  : Variable and Systematic error removed data, C

**Procedure** : S = Extract fixation data from raw eye gaze data, S

      Variable Error Correction:

      *S*_*t*_ = GSPfiltering(*S*_*x*_, *S*_*y*_)

$\hat{R_{k}}$ = KalmanSmoothing(*S*_*t*_*x*__, *S*_*t*_*y*__)

    Systematic error removal:

      Obtain 1 *T* derived from 1 nearest calibration point

      Obtain corrected data *C*, by transforming the $\hat{R_{k}}$ using *T* as,

$C=\hat{R_{k}}*T$

**Algorithm 4**: Pseudocode for Path B

**Input**   : Raw Gaze data, *S* = (*S*_*x*_, *S*_*y*_)

**Output**  : Variable and Systematic error removed data, C

**Procedure** : S = Extract fixation data from raw eye gaze data, S

     Variable Error Correction:

     *S*_*t*_ = GSPfiltering(*S*_*x*_, *S*_*y*_)

$\hat{R_{k}}$ = KalmanSmoothing(*S*_*t*_*x*__, *S*_*t*_*y*__)

    Systematic error removal:

     Obtain 4 *T*s derived from 4 nearest calibration points

     Find weighted *T* from the 4 *T*s using inverse weighing function

     Obtain corrected data *C*, by transforming the $\hat{R_{k}}$ using *T* as,

$C=\hat{R_{k}}*T$

#### 4.3.2 Unsupervised approach—Paths C and D

This approach is based on the ‘*n*—nearest stimulus point’ (not the calibration point), in contrast to the ‘required fixation location’ [12], ‘probable fixation location’ [39], and ‘closest stimulus point’ [14]. In our case, *n* = 2 or 4 neighboring stimulus points.

**Fig 8:**
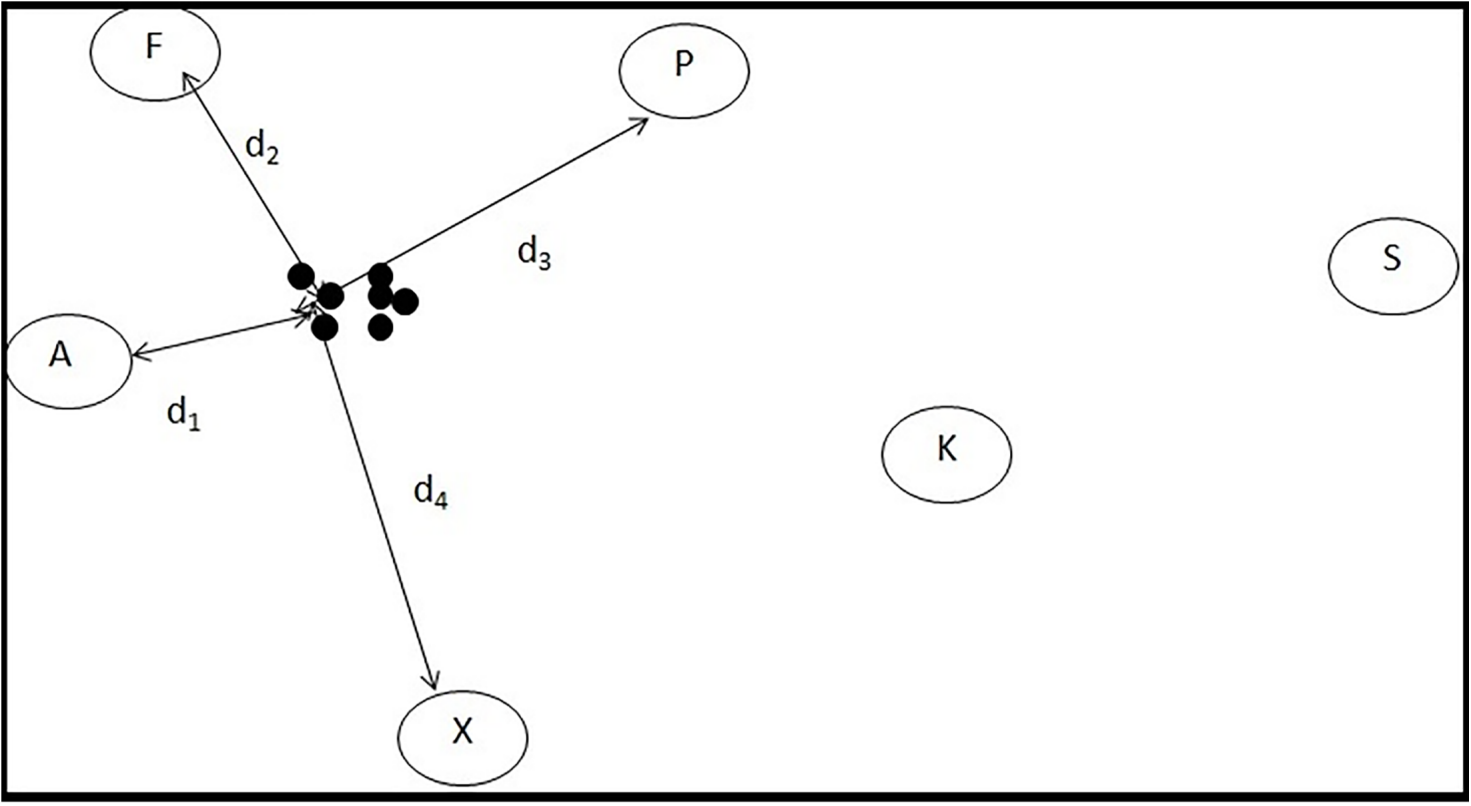
Demonstration of inverse weighing function for 4 nearest neighbor stimuli points.

For path C, as shown in Fig 7, 4 nearest stimulus points are selected and transformation matrices with respect to each of these 4 locations are derived. Later, inverse weighing function is applied, as discussed in Eq (16), to get the corrected data. For path D, as shown in Fig 7, similar approach is applied but with only 2 nearest stimulus points instead of 4 (Fig 8). For the fixation data represented by black dots, the 4 nearest stimuli are A, F, P and X with the Euclidean distances *d*_1_, *d*_2_, *d*_3_ and *d*_4_ from the fixation center. The weights are chosen to be inversely proportional to the distance, i.e. lesser the distance, larger the weight; which implies that the corrected fixation would be more biased towards the nearer neighbors. In the unsupervised approach, we have used 4 and 2 nearest stimulus points. The usage of 4 points is analogous to the one mentioned in supervised approach. The psuedocodes for paths C and D are provided in Algorithms 5 and 6, respectively.

**Algorithm 5**: Pseudocode for Path C

**Input**   : Raw Gaze data, *S* = (*S*_*x*_, *S*_*y*_)

**Output**  : Variable and Systematic error removed data, C

**Procedure**: S = Extract fixation data from raw eye gaze data, S

      Variable Error Correction:

      *S*_*t*_ = GSPfiltering(*S*_*x*_, *S*_*y*_)

$\hat{R_{k}}$ = KalmanSmoothing(*S*_*t*_*x*__, *S*_*t*_*y*__)

     Systematic error removal:

      Obtain 4 *T*s derived from 4 nearest stimulus points

      Find weighted *T* from the 4 *T*s using inverse weighing function

      Obtain corrected data *C*, by transforming the $\hat{R_{k}}$ using *T* as,

$C=\hat{R_{k}}*T$

**Algorithm 6**: Pseudocode for Path D

**Input**   : Raw Gaze data, *S* = (*S*_*x*_, *S*_*y*_)

**Output**  : Variable and Systematic error removed data, C

**Procedure**: S = Extract fixation data from raw eye gaze data, S

     Variable Error Correction:

     *S*_*t*_ = GSPfiltering(*S*_*x*_, *S*_*y*_)

$\hat{R_{k}}$ = KalmanSmoothing(*S*_*t*_*x*__, *S*_*t*_*y*__)

    Systematic error removal:

     Obtain 2 *T*s derived from 2 nearest stimulus points

     Find weighted *T* from the 2 *T*s using inverse weighing function

     Obtain corrected data *C*, by transforming the $\hat{R_{k}}$ using *T* as,

$C=\hat{R_{k}}*T$

From the above discussion it is clear that unsupervised approaches mainly aim on dragging the gaze data towards its nearest stimulus, whereas, supervised approaches handle the gaze data by considering the direction and magnitude of the systematic error as obtained in the calibration phase. However, usage of any one of this method solely cannot serve all the types of stimulus. For instance, if the stimulus points are very densely packed (e.g. designed stimulus Recall-Recognition (RR) task with more than 24 words), then the nearest stimulus-based noise cleaning fails considerably. In such cases, the supervised approaches can prove to be beneficial. In contrast to this, in case of stimulus points being placed far apart (e.g. designed stimulus Number Gazing (NG) task), the gaze data could be handled very well using the nearest stimulus positions. Hence, the accuracy of noise cleaning relies on the nature of the stimulus and so we have experiemented with both the supervised and unsupervised approaches.

It is to be noted that the four paths A, B, C and D are independent of each other and we tested them one after the other on the data to check the effectiveness of each of them.

## 5 Experimental paradigm

This section discusses the experimental setup, various stimuli used and the details of the data collection procedure adopted in the present study.

### 5.1 Setup

**Fig 9:**
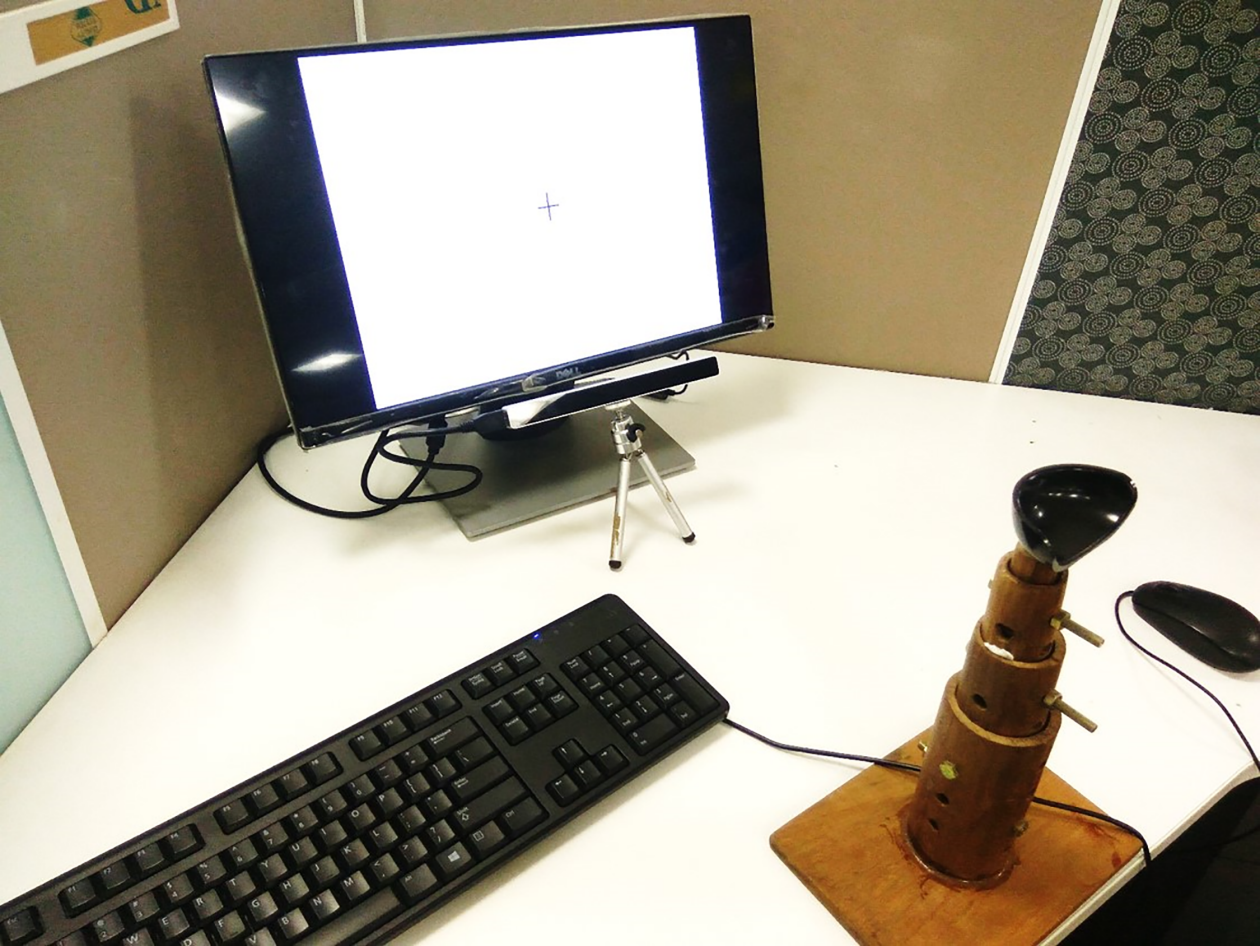
Experimental setup with the eye tracker at the bottom of the display and a chin rest.

The experimental setup is shown in Fig 9. We have used a low-cost eye tracker from Eye Tribe [59] having a sampling rate of 30 Hz. The Eye Tribe device is placed below the screen as shown in Fig 9. An wooden chinrest fixed on the table is used while collecting the eye gaze data. A height adjustable chair was used during data collection. The stimulus is shown on a computer screen (1366 × 768) placed at a distance of approximately 60 cm from the participants. The entire experiment is carried out in a closed, quite room under constant lighting conditions.

### 5.2 Participants

Twenty participants (12 females and 8 males, mean age 32 ± 5.54 years) are selected from our research lab for the experimentation. All of them had normal or corrected to normal vision with spectacles. We have ensured that they belong to similar cultural backgrounds and have similar educational qualification. None of them had any background history of any mental or physical ailments. Participants are allowed to wear spectacle during data collection. The clearance on ethical issues for handling and analysis of the data collected has been acquired from Institutional Review Board of Tata Consultancy Services Ltd. (TCS). Informed consent is also taken from the participants and the data is anonymized for further processing.

### 5.3 Data collection

The experimental protocol and the tasks to be performed are explained to the participants before starting the experiment. The participants are asked to sit comfortably on a chair with adjustable height and a chin-rest is used to minimize the head movements. The participants then performed an initial software development kit (SDK)-based calibration (provided by the Eye Tribe sensor). The goodness of calibration is represented on a scale of 1-5. A score of 5 corresponds to best calibration giving an error below 0.5 degree, whereas the error is more than 1.5 degrees for the score of 2. Score 1 indicates the calibration is extremely bad and re-calibration needs to be performed. Before starting the actual experiment, the participants are encouraged to take part in a practice session in order to have a better understanding of the stimulus and the task to be performed. The stimulus used for practice sessions are similar to that used for the actual experimentation, however, not exactly the same in terms of the set of words to be recalled or the numbers to be gazed. The data collection is carried out in 2 phases as described in the following subsections.

#### 5.3.1 Phase one: Initial calibration

An initial calibration (both SDK-based and our designed one) is performed once for a single (first) participant and the calibration results are applied on the remaining participants. The position of the chin rest and the eye tracker are not altered for the remaining participants. Next participant onwards, 2 tests (NG and the RR task) are performed as explained earlier. Corresponding eye gaze data are collected and are used for further analysis.

#### 5.3.2 Phase two: Repeated calibration

The accuracy of the protocol is later tested for ‘multiple time’ or ‘repeated calibration’, against one time calibration. Here, every participant performs both the SDK and the designed calibration, before every test session.

Participants signed a consent form before the commencement of the experimentation. The data corresponding to every participant is anonymized.

## 6 Results and discussion

The performance evaluation of our proposed methods are done in terms of algorithm/approaches for the following scenarios

1. variable error removal techniques
2. comparison of supervised and unsupervised approaches for systematic error removal
3. comparison of single calibration against multiple calibration protocols
4. evaluation of proposed noise removal method for long duration tasks

**Fig 10:**
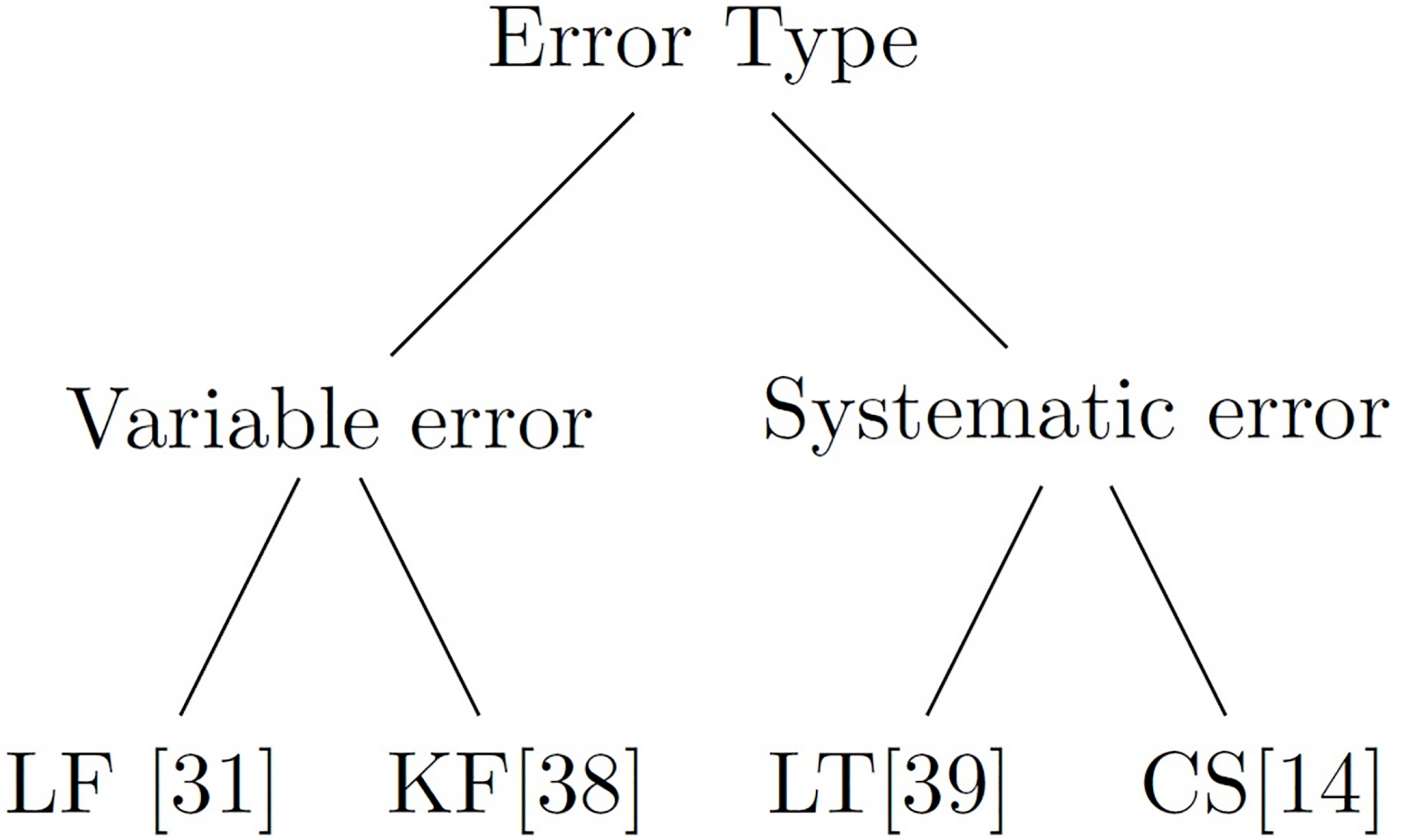
State of the art methods considered for comparison for different types of error, where, LF = Low pass filtering, KF = Kalman filtering, LT = Linear Transformation and CS = Closest Stimulus based approach.

We have also compared our designed approaches with the closely related state of the art methods as explained in the tree diagram given in Fig 10.

### 6.1 Variable error removal technique

The performance evaluation is carried out to test if our proposed method is able to extract the desired dense cluster of input fixation points.

**Fig 11:**
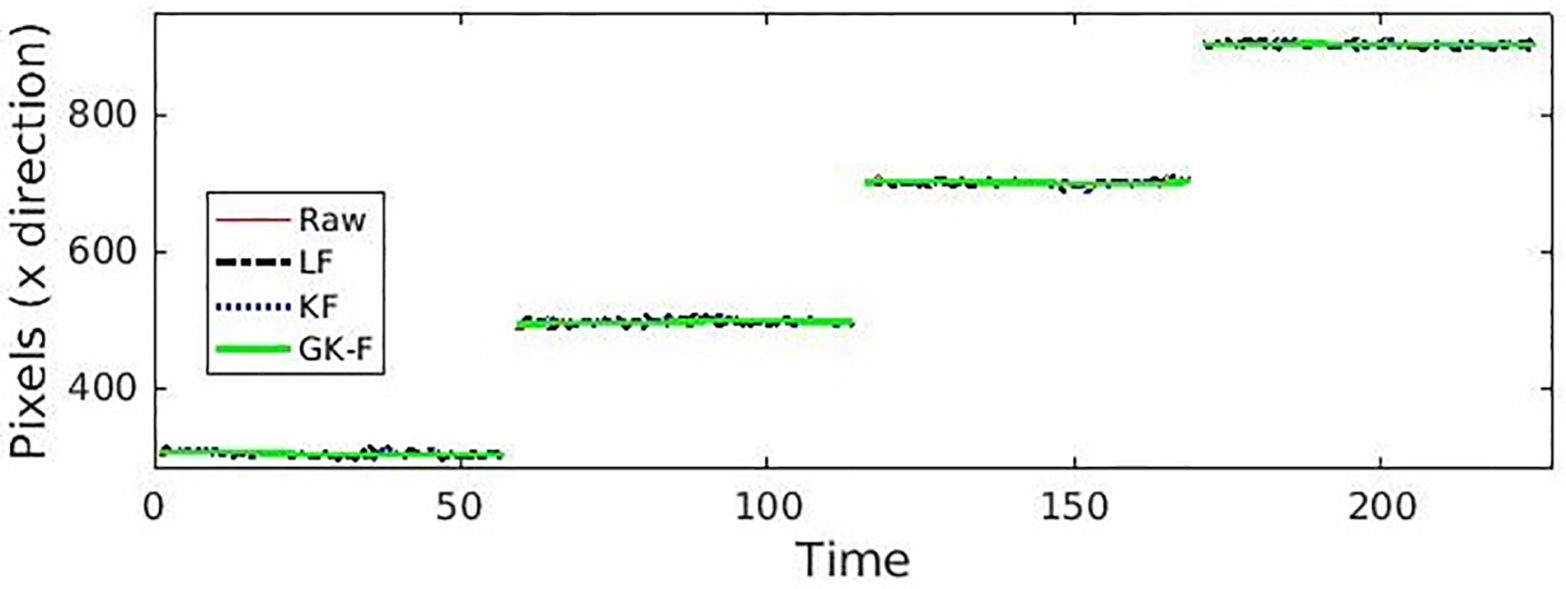
Comparison of different filtering approaches for the NG task wherein the participant gazed at 4 different numbers. Here, LF = Low pass filtering, KF = Kalman filtering, GSP + KF = Graph signal processing and Kalman filter.

Hence, by considering the problem associated with the variable errors, our proposed method has been compared with most widely used filtering approaches for eye tracking, i.e., Low pass filtering and Kalman Filter. Fig 11 shows the effects of different filtering approaches used on the gaze data corresponding to the NG task.

**Fig 12:**
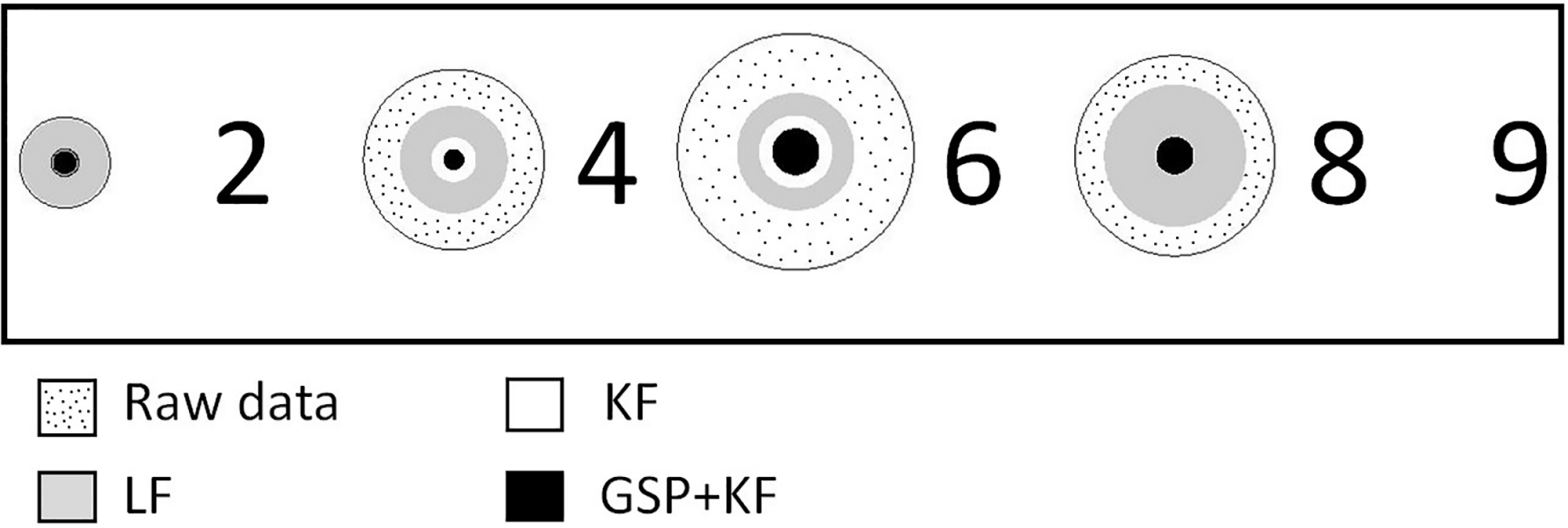
Demonstration of different filtering approaches in terms of smoothness for NG task. Note that for the gaze chunk on the digit ‘1’, the values of *SR* in terms of degrees are 0.932°, 0.92°, 0.26°, 0.2°, respectively for raw data, LF, KF and GSP + KF approaches. Here, LF = Low pass filtering, KF = Kalman filtering, GSP + KF = Graph signal processing and Kalman filter.

Fig 12 shows the sample results of different filters for the NG task for one particular participant (assuming systematic error to be zero). The participant is asked to gaze the numbers, 1-3-5-7. The radius of each circle reflects the general smoothness of the data. Smaller the radius of the circle, better is the filtering capabilities. For the gazed number ‘1’ in the Fig 12, the radius of raw data, low pass filter (LF) and Kalman filter (KF) filtered data is almost the same and hence, the circles are overlapping. Similarly, for the gazed number 7, the radius of Kalman filter KF filtered data is slightly larger than the GSP + KF filtered data. Note that the radii of the proposed GSP + KF data chunk are least for all the gazed numbers.

In order to get insight of the change in the radius from raw to filtered data, we have defined Smoothness Ratio (SR) metric as the ratio of the radius of raw data chunk *D*_*r*_ and the radius of the filtered data *D*_*f*_ (Eq 17). Fig 12 depicts the visualization of filtered output assuming systematic error as zero.

$$\begin{array}{c} SR=\frac{D_{r}}{D_{f}} \end{array}$$ (17)

**Fig 13:**
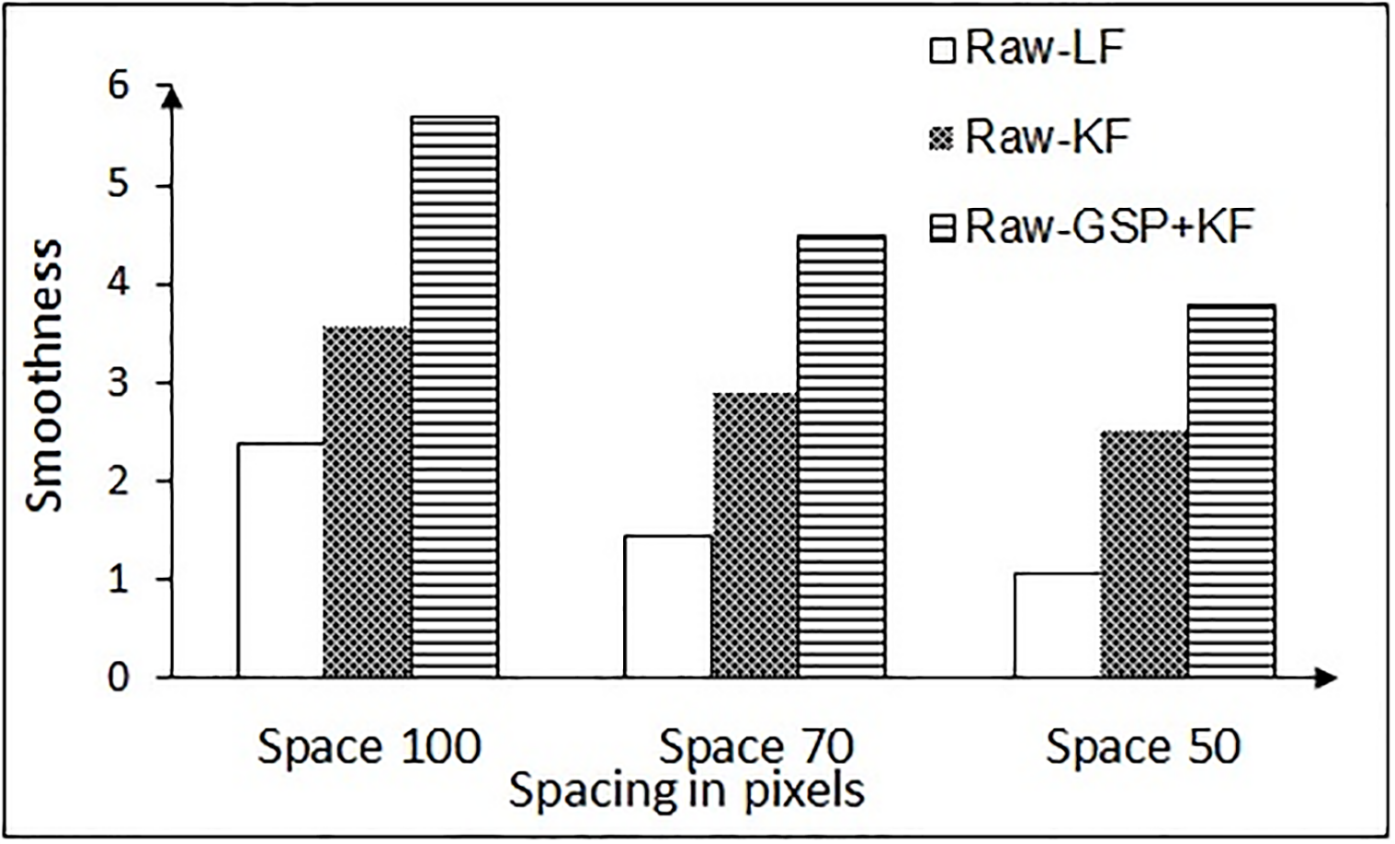
Smoothness ratio of proposed and existing methods in NG task. Here, LF = Low pass filtering, KF = Kalman filtering, GSP + KF = Graph signal processing and Kalman filter.

**Fig 14:**
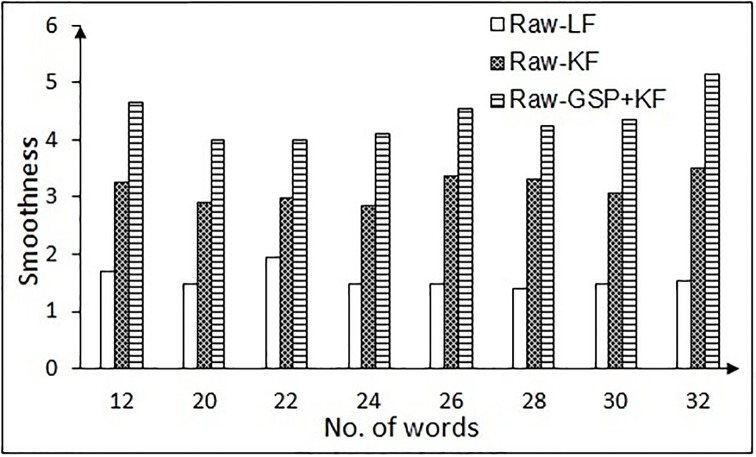
Smoothness ratio of proposed and existing methods in the RR task. Here, LF = Low pass filtering, KF = Kalman filtering, GSP + KF = Graph signal processing and Kalman filter.

Larger values of SR mean better the filtering approach. The estimated SR values for each filter on both the tasks are shown in Figs 13 and 14. Fig 13 shows the SR in the NG task, when the test is carried on the different categories of word spacing in comparison to the existing methods. Fig 13 clearly depicts that the performance of GSP + KF is by far better than the existing methods. There is an enhancement of 69% over the complete spacing against Raw-LF and more than 27% against Raw-KF (raw means data taken directly from the eye tracker device). SR in recall-recognition (RR) task for the proposed and existing methods is shown in Fig 14. The enhancement ranges from 56% (for minimum words) to 66% (for maximum words) compared to Raw-LF. It is to be noticed that, even though the number of words increases, the performance of the proposed method is still better.

The variable error also arises due to eye tracker hardware, exhaustion or fatigue of the user, etc. Thus a closeness measure (CL) is also required to know how the points deviate or spread across its cluster center. We have computed the *CL* of the data chunk with respect to its cluster center as given by Eq (18),

$$\begin{array}{c} CL=\sqrt{\frac{\sum_{i=1}^{N}{\left(p^{`}-p_{i}\right)}^{2}}{N}} \end{array}$$ (18)

where *N* is the number of samples, *p* = (*x*, *y*) are the fixation data coordinates and $p^{`}=\left(x^{`},y^{`}\right)$ represents the coordinates of the cluster center. We call this metric as the *closeness* *measure*, as it computes the distance of cluster center from rest of the points. Lower closeness values indicate better filtering approach.

**Fig 15:**
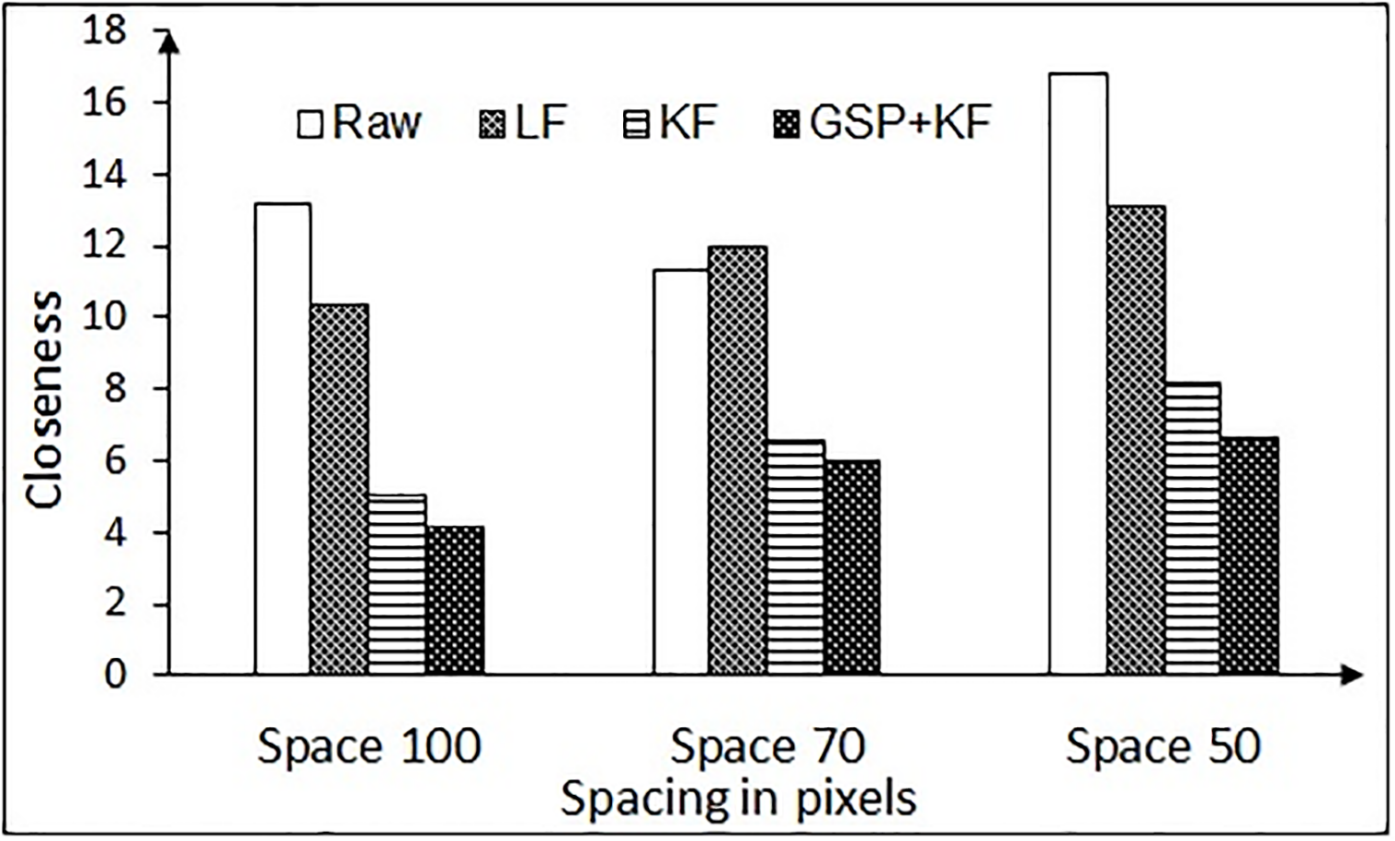
Closeness measure results for variable error correction of the NG task for different spacing (for one-time calibration protocol). Here, LF = Low pass filtering, KF = Kalman filtering, GSP + KF = Graph signal processing and Kalman filter.

**Fig 16:**
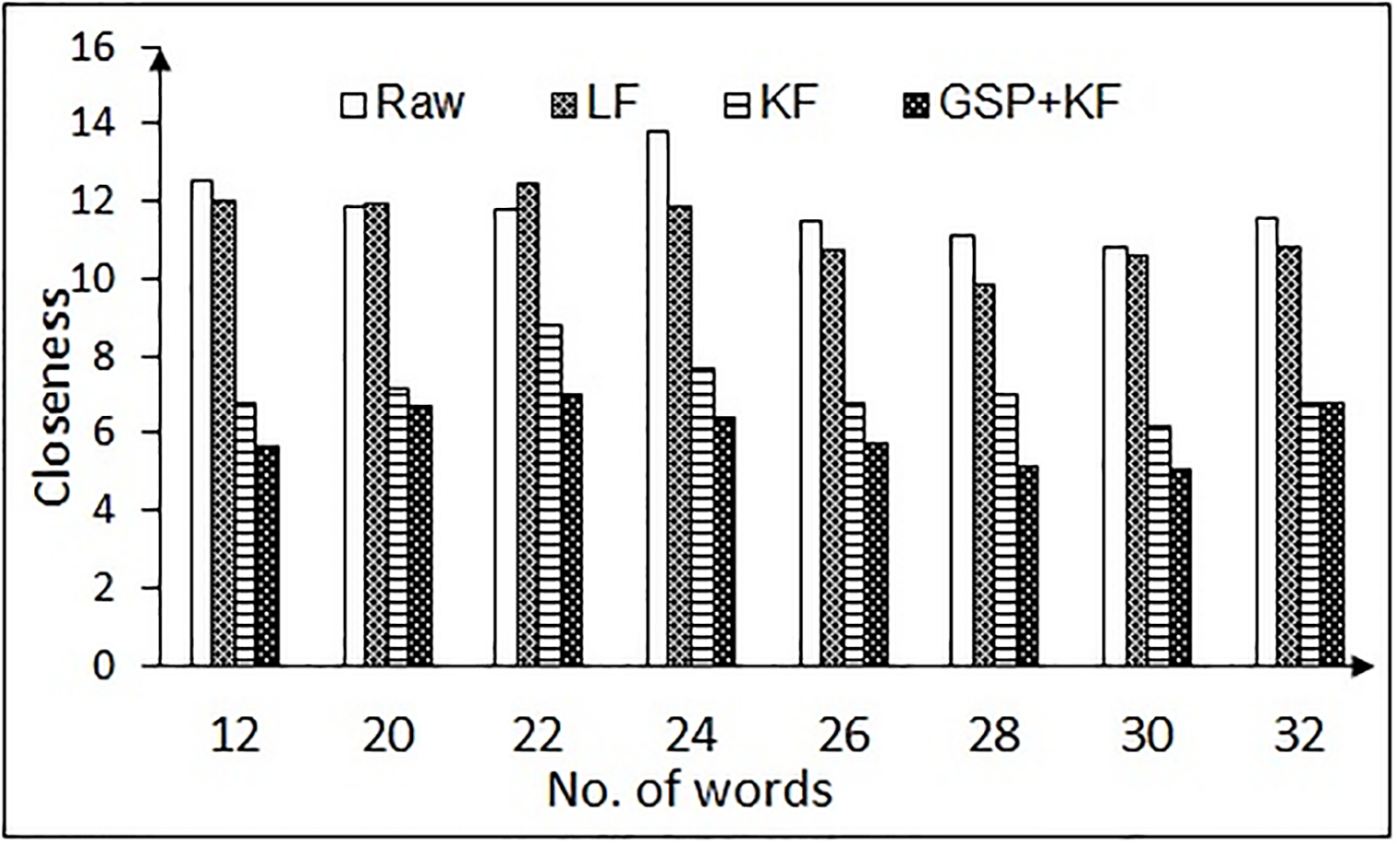
Closeness measure results for variable error correction of the RR task for different number of words (for one-time calibration protocol). Here, LF = Low pass filtering, KF = Kalman filtering, GSP + KF = Graph signal processing and Kalman filter.

The results for variable error removal, based on closeness measure are presented in Figs 15 and 16, for the NG task and the RR task, respectively. It is to be noted that the combination of graph signal processing and Kalman filter performs better in comparison to the low pass filter and Kalman filter in increasing the compactness in the data chunk. Hence, this combination is used for the further analysis.

Note that in case of the NG task, the number of participants are 20, out of which for the first 10, we manually selected the chunks in the gaze data. From these chunks, the data of window length 2 seconds was taken for further analysis because as per the subjective feedback, gazing on a particular entity for more time is difficult, which would rather encompass micro-saccades. For remaining 10 participants, we introduced a ‘click’ event in the NG task and the data of window length 2 seconds before the click event was considered. This was done to verify any significant change in the performance of the algorithms due to click event and it was seen that there was no significant effect. Hence, for the rest of the stimulus types, i.e. for the NG task with multiple calibration and modified NG task for long duration analysis, the click event was used to speed up the process.

### 6.2 Comparison of unsupervised and supervised approach for systematic error removal

**Fig 17:**
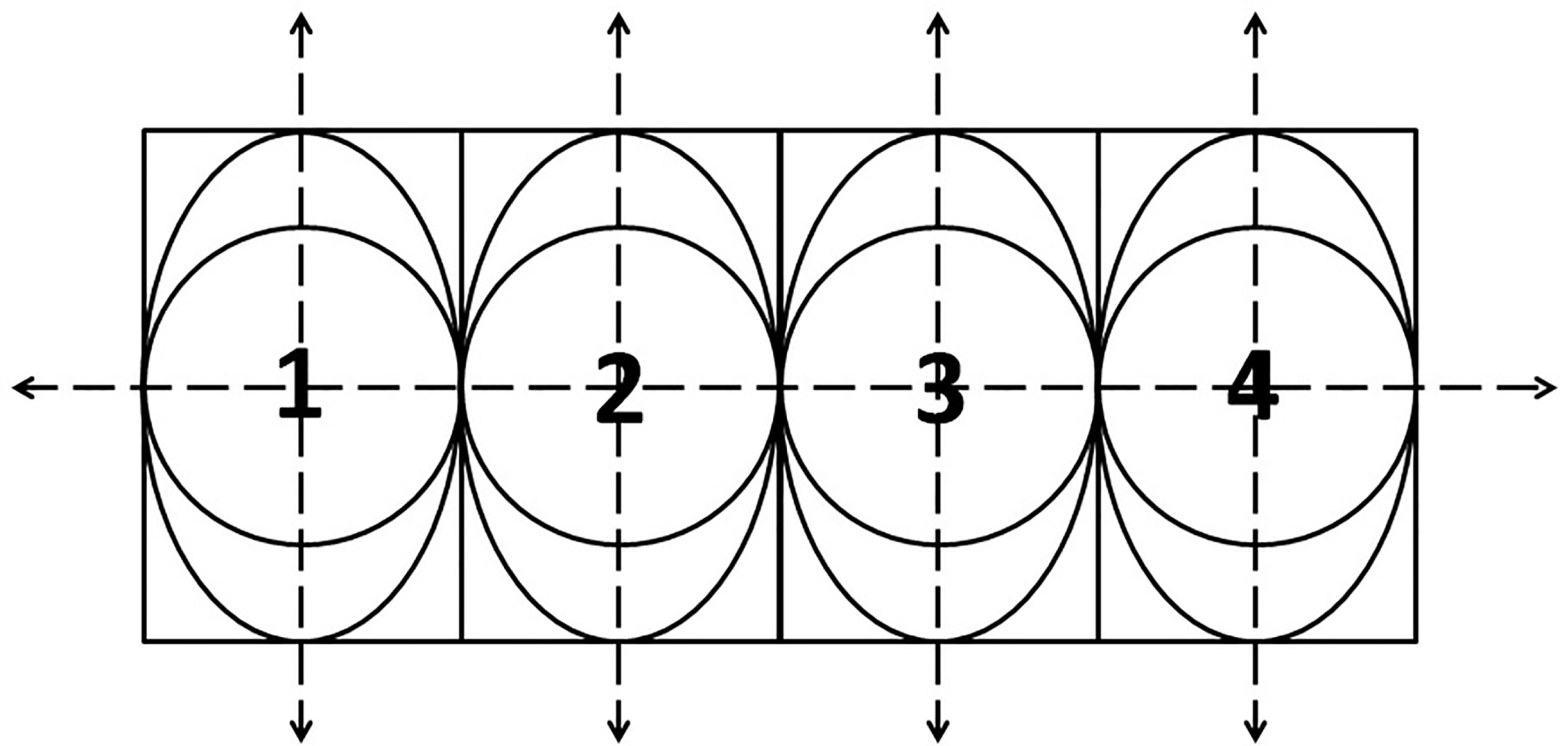
Pictorial scheme of different types of boundaries defined around each number in the NG task.

For systematic error removal, the approach used to measure the algorithmic performance is through the accuracy of having the centroid of the gaze chunk in the area of interest (AOI) of the stimulus. Hence, efficient designing of the AOI boundary also plays a vital role in the computation of accuracy. Based on this, three different AOIs are considered, viz. circular, rectangular and elliptical, as shown in Fig 17 for the NG task. The accuracy thus obtained for raw gaze data is reported in Table 2. The reason for using the raw gaze data in this context is to throw light on the effectiveness of the boundaries in the absence of any noise cleaning techniques. It is evident from Table 2 that rectangular boundary provides maximum accuracy and hence, further analysis is carried out using the rectangular boundary only.

**Table 2:** Accuracy (%) of detecting the gazed number using the 3 different boundaries for raw gaze data.

AOI	100 Space	70 Space	50 Space
**Circle**	57.5	53.75	38.75
**Ellipse**	66.25	65	57.5
**Rectangle**	71.25	67.5	60

**Fig 18:**
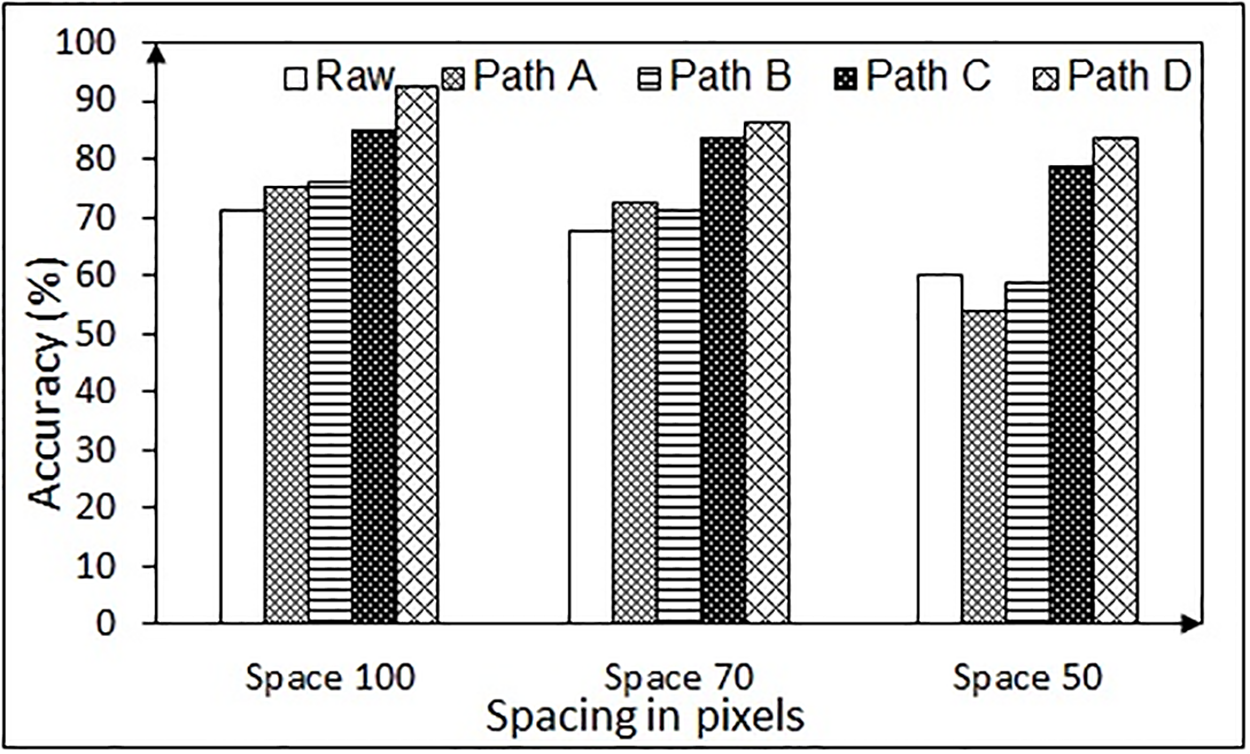
Accuracy of detecting the gazed numbers using different algorithmic chains for the NG task (for one-time calibration protocol).

**Fig 19:**
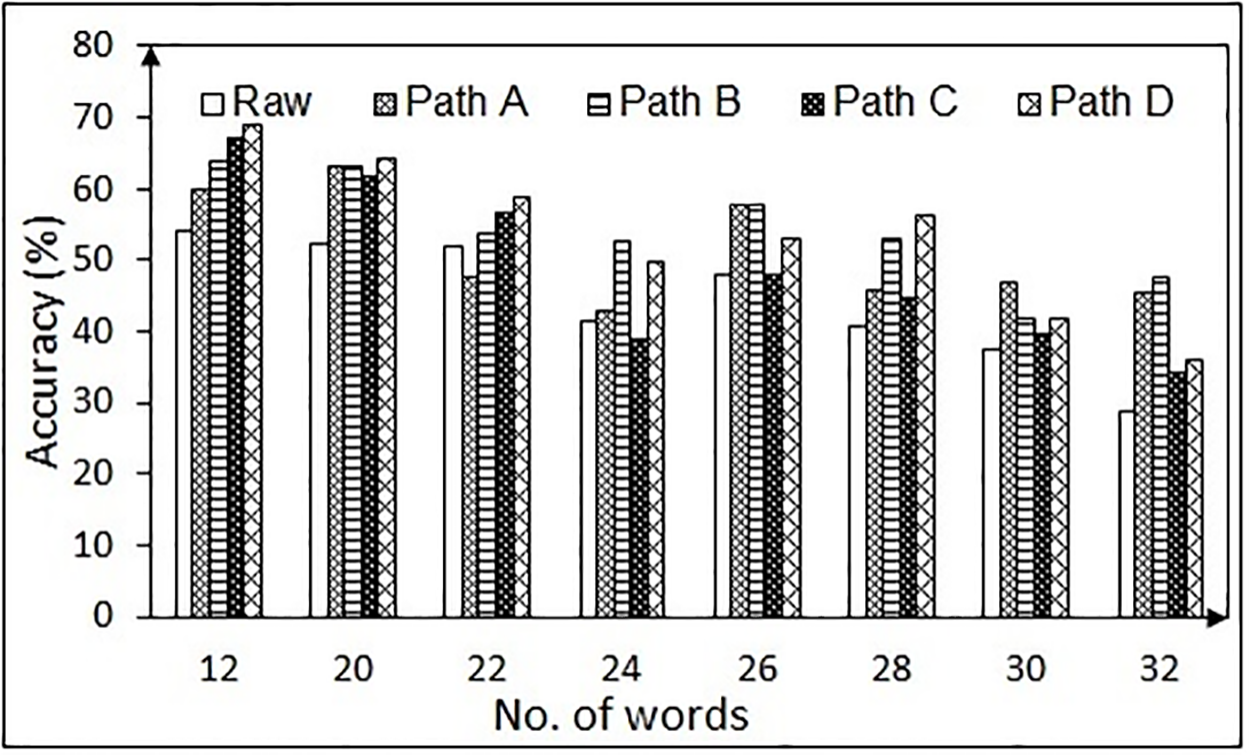
Accuracy of detecting the gazed words using different algorithmic chains in the RR task (for one-time calibration protocol).

Next the performance of each path (Path A through D as depicted in Figs 6 and 7) is assessed in terms of accuracy for the rectangular boundary and is shown in Figs 18 and 19, for the NG task and the RR task, respectively. In case of NG task, the accuracy of the raw data decreases considerably with the decrease in inter-number spacing. However, with the proposed approach, the error is reduced, thereby enhancing the accuracy. For the RR task, the accuracy of all the approaches drops with the increase in number of words (thereby decrease in spacing between the neighboring words). However, it is to be noted that it is the best possible option to use 12 words in order to get good accuracy in such systems. The results confirm that even with one-time calibration, the designed algorithmic chain can handle the variations in the gaze data due to subject-specific differences, making it a practical solution for patients who are unable to perform calibration.

### 6.3 Comparison of single calibration against multiple calibration methods

We have further compared the proposed and the existing noise cleaning methods considering multiple calibration (i.e. calibration for each participant) and proposed one time calibration. The stimulus chosen for comparison are NG task with 50 pixels (inter-digit spacing) and RR task with 32 words (i.e. 16 words/column). The comparison of accuracy of handling the systematic error for single and multiple calibration is given in Table 3. The nature of systematic error is calibration-dependent, which is evident from this table. Also, with multiple calibration, the accuracy of detecting the desired stimulus point increases, however, the difference in accuracy for both the cases is not considerably high. Hence, in cases where multiple calibration is not possible, it is acceptable to go ahead with single calibration, provided the eye tracker and chin rest positions are not altered.

**Table 3:** Comparison of average accuracy (%) in systematic error correction for different calibration protocol (RR-Recall recognition, NG-Number gaze).

Task	Calibration Type	Raw	Path A	Path B	Path C	Path D [39]	LT	Closest Stimulus [14]
**RR**	**Single**	28.86	45.38	**47.47**	34.07	36.16	18.6	38.24
	**Multiple**	38.33	**40**	38.33	36.67	38.33	10	36.67
**NG**	**Single**	60	53.75	58.75	78.75	**83.75**	78.75	88.75
	**Multiple**	70	82.5	82.5	87.5	**90**	82.5	100

In Table 3 it can be seen that the overall accuracy for the supervised approach (for RR task) for multiple calibration is lesser in comparison to its single calibration counterpart; in contrast to the NG task where the reverse behavior is seen. This can be attributed to the fact that not all the participants were able to perform the calibration phase properly, thereby degrading the overall accuracy. This behavior is seen specifically for the RR task as the stimulus points (words) are densely packed in this case, hence, the effect of the *n*-nearest calibration points for the supervised approach has more impact which might be degraded due to bad calibration. In case of the NG task, the calibration points at the top of the screen had more impact owing to the placement of the numbers on the screen.

Table 3 shows the comparison of various proposed approaches over the closely related state-of-the-art methods. It is to be noted that in case of NG task, the proposed unsupervised approach (Path C and D of Fig 7) outperforms [39]. It is also evident from the Table 3 that though the method proposed in [14] outperforms all other methods for NG task, but it does not work in cases where the number of stimuli points is large or the stimuli are densely packed. Hence, the results obtained using [14] is not good for the RR task. The main reason is that the method in [14] is based on closest stimulus point and hence, the accuracy is computed by looking at the closest stimulus point from the gaze chunk obtained using mean shift algorithm [14]. In our case, the error free data either lies in the correct stimulus region {*R*_*c*_ ∈ *U*}, wrong stimulus region {*R*_*w*_ ∈ *U*} or in no-man’s land {*R*_*n*_ ∈ *U*, where *R*_*n*_ ∉ (*R*_*c*_ ∪ *R*_*w*_)}; due to the rectangular boundary defined around each stimulus point, where *U* corresponds to the overall screen region. However, it is to be noted that the method given in [14], forcibly moves a fixation to the closest point and hence, a true fixation away from the stimulus is not detected by the algorithm. Therefore, all our proposed methods are performing better than existing methods as reported in Table 3, maximum accuracy is obtained with Path B for RR task and that for NG task is obtained through Path D.

### 6.4 Performance of proposed noise removal methods for long duration tasks

**Fig 20:**
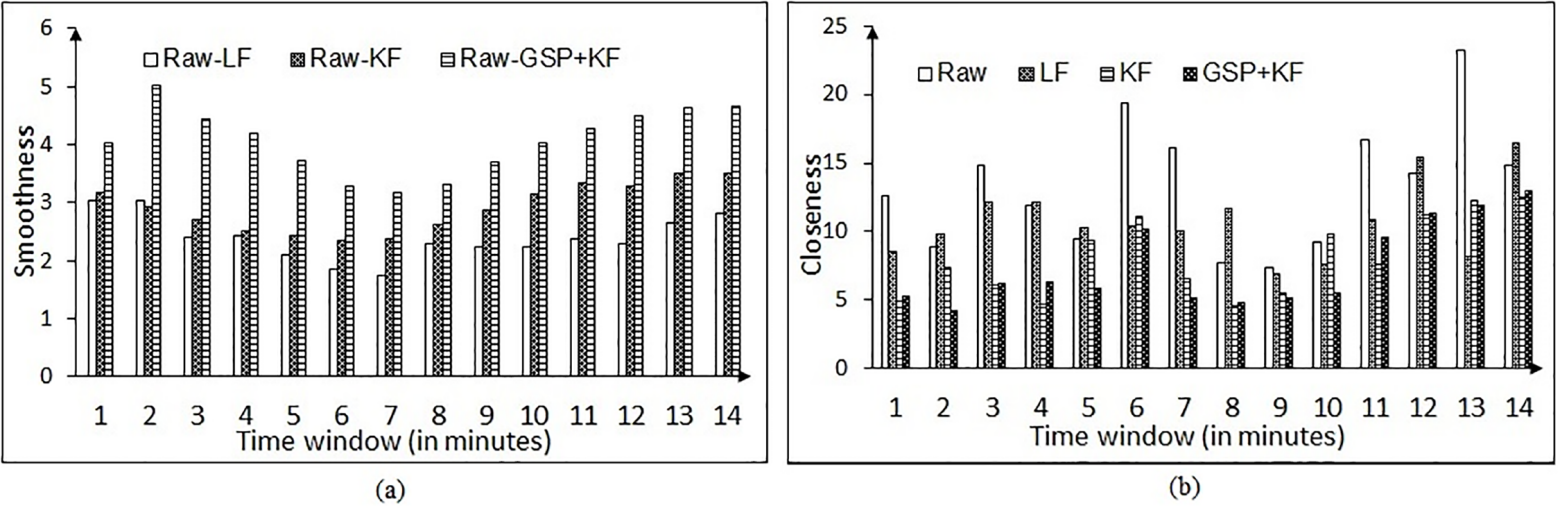
Removal of variable error for participant P1. (a) Smoothness parameter, (b) Closeness parameter.

To check the scalability aspects for long duration task on the proposed eye tracking noise removal methods, the NG task is modified. The inter-number spacing is selected to be 50 pixels (as this is the least spacing). The total duration of the task is set to 15 minutes approximately in which 9 random single digit numbers (4 odd numbers) appear on the screen at a time. The participants are expected to gaze and click on these 4 odd numbers only. After 4 clicks a new set of numbers appears on the screen. Totally 3 participants were taken for this case. Fig 20 shows the variable error related parameters-smoothness and closeness for one participant P1.

**Fig 21:**
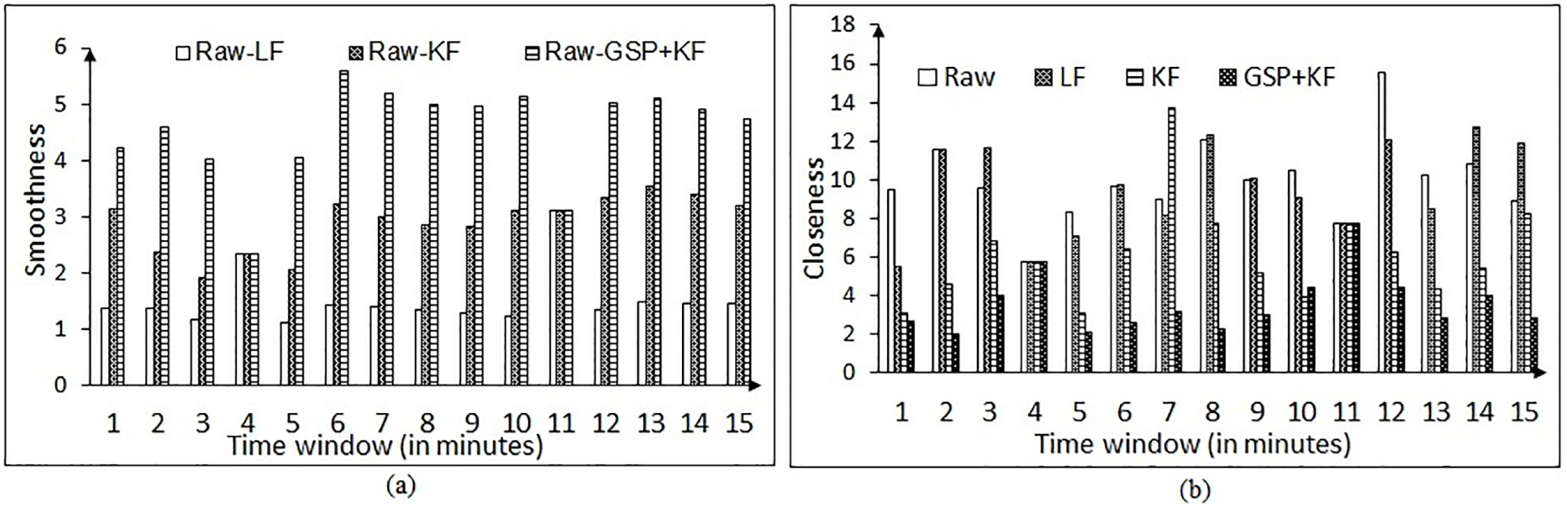
Removal of variable error for participant P2. (a) Smoothness parameter, (b) Closeness parameter.

The participant P1 performed the task for a duration of 15 minutes approximately and hence, the results are shown by computing the parameters over a window length of 60 seconds. It is noted from Fig 20 that our variable error removal technique is able to reduce the noise (i.e. extracting dense fixation chunk from raw eye tracker data), whereas, the parameters corresponding to the raw data degrade considerably over time as the participant felt exhausted and stopped the experiment abruptly at the 14^th^ minute. This observation (i.e. how subjective fatigue and exhaustion affects the variable error) also emphasizes the fact reported in [15]. In order to establish the fatigue factor on variable error, we have carried out same analysis for another participant P2 who was well-acquainted with the data capture procedures as he had participated several times during the initial phases of our experiment. From the subjective feedback, it was clear that he did not feel exhausted during the study and Fig 21 also supports the fact. The nature of variable error (see Fig 21) is somewhat constant owing to the raw data, whilst the proposed GSP + KF method is successful in handling the variable noise in contrast to the state-of-the-art methods.

Figs 20 and 21 truly justify how the proposed method is robust enough in handling the variable error induced by fatigue, head movement and exhaustion etc. Table 4 provides the consolidated results for the variable error correction with respect to the closeness and smoothness parameters for the proposed GSP + KF technique. It can be seen that the variation in the parameters for long duration task is within the 10% range of the short duration task. Table 5 shows the accuracy of correcting the systematic error for the short and long duration task. Note that the accuracies are consistent bereft of the duration of the task while handling the systematic error. Path C and D perform better in denoising the systematic error.

**Table 4:** Comparison of variable error correction in terms of closeness measure and smoothness ratio in short and long duration task using GSP + KF.

	Short Duration Task	Long Duration Task
**Smoothness**	3.79	3.96
**Closeness**	6.62	5.36

**Table 5:** Comparison of average accuracy (%) in systematic error correction for short and long duration task.

	Short Duration Task	Long Duration Task
**Path A**	53.75	53.95
**Path B**	58.75	54.78
**Path C**	78.75	74.49
**Path D**	83.75	73.71

## 7 Conclusions

The study aims at denoising a low-cost eye tracker in order to make it a perfect choice for the applications, such as rehabilitation, cognitive assessments, etc. The noise characteristics of a low resolution eye tracker are studied thoroughly and optimized approaches are designed to handle the errors associated with those errors. The algorithms are tested on 2 simple test stimuli and it is seen that our approach improves the overall performance of the system. In case of variable error, our proposed method reduces the dispersion of data points (i.e. closeness) by 48.98% and 59.53% in comparison with the raw data, for RR and the NG task, respectively. For systematic error removal, the results show improvements of about 17.86% and 15.25% over the raw data, for RR and NG tasks (taken average across all proposed paths for one time calibration). The chosen test stimuli are closely related to the psychological tests and our results are motivating enough for the usage of eye tracker as a physiological sensor that can be used to extract more subject specific information such as working memory, attention or engagement, visual-motor coordination, etc., in real-time feedback for home-based applications. In addition to this, we have devised a one-time calibration protocol to avoid repeated calibration. Results confirm that the proposed approach gives satisfactory results in comparison to its multiple calibration scheme. Thus, it can be used successfully for patients who are unable to perform calibration due to some medical conditions. We have also evaluated our algorithms for long duration tasks and the results obtained are quite satisfactory. The system suits well for rehabilitation purpose. For the sake of rigorous scientific applications, the study needs to be further examined with detailed case studies governing the cognitive and behavioral aspects of eye movements research. In future we intend to increase the task duration further and study the effects on applications involving dynamic visual scenes like that of driving scenarios.
